# Supplementary material for: Reduced Atlantic reef growth past 2 °C warming amplifies sea-level impacts
Source: Nature. 2025 Sep 17;646(8085):619–26. doi: 10.1038/s41586-025-09439-4 (PMC12527930; doi:10.1038/s41586-025-09439-4)
Supplement: Supplementary file 1 — Supplementary Tables 1–8. [file 41586_2025_9439_MOESM1_ESM.pdf]

nature portfolio

https://doi.org/10.1038/s41586-025-09439-4

Supplementary information

Reduced Atlantic reef growth past 2 °C warming amplifies sea-level impacts

In the format provided by the authors and unedited

SI Guide: Reduced Atlantic reef growth past 2°C warming amplifies sea-level impacts

Chris T. Perry[1*], Didier de Bakker[1], Alice Webb[1], Steeve Comeau[2], Ben Harvey[3], Christopher E. Cornwall[4], Lorenzo Alvarez-Filip[5], Esmerelda Pérez-Cervantes[5], John Morris[6], Ian Enochs[6,7], Lauren T. Toth[8], Aaron O'Dea[9,10], Erin Dillon[9], Erik Meesters[11], William F Precht[12]

[1] Geography, Faculty of Environment, Science and Economy, University of Exeter, Exeter, UK
[2] Sorbonne Université, CNRS-INSU, Laboratoire d'Océanographie de Villefranche, 181 chemin du Lazaret, F–06230 Villefranche-sur-Mer, France
[3] Shimoda Marine Research Center, University of Tsukuba, Shimoda, Shizuoka, Japan
[4] School of Biological Sciences, Victoria University of Wellington, Kelburn 6140, Wellington, New Zealand
[5] Biodiversity and Reef Conservation Laboratory, Unidad Académica de Sistemas Arrecifales, Instituto de Ciencias del Mar y Limnología, Universidad Nacional Autonoma de México, Puerto Morelos, Quintana Roo, Mexico
[6] Cooperative Institute for Marine and Atmospheric Studies, University of Miami, Miami, Florida, USA
[7] Atlantic Oceanographic and Meteorological Laboratory, Ocean Chemistry and Ecosystem Division, NOAA, Miami, Florida, USA
[8] U.S. Geological Survey, St. Petersburg Coastal and Marine Science Center, St. Petersburg, Florida, USA
[9] Smithsonian Tropical Research Institute, Balboa, Republic of Panamá
[10] Sistema Nacional de Investigación, SENACYT, Republic of Panamá
[11] Wageningen Marine Research (WMR), Wageningen University & Research, Den Helder, Netherlands
[12] Bio-Tech Consulting, Coastal and Marine Sciences, Miami Lakes, Florida, USA

* Corresponding author. E-mail: c.perry@exeter.ac.uk

SI Table 1. Shared Socioeconomic Pathways (SSP) scenarios considered in this study and aligned projected global temperature trends from the IPCC 6th Assessment Report (AR6) (Fox-Kemper et al. 2021).

Scenario	Global carbon emission trends	Projected (90% probability) globally averaged surface air temperature increase over the period 2081–2100 compared to 1850-1900
SSP1–2 .6	SSP1–2 .6 stays below 2.0°C warming relative to 1850-1900 (median) with implied net zero emissions in the second half of the century.	1.3°C–2.4°C
SSP2–4.5	SSP2–4.5 is approximately in line with the upper end of aggregate Nationally Determined Contribution emission levels by 2030. The SSP2-4.5 scenario deviates mildly from a 'no-additional- climate-policy' reference scenario, resulting in a best-estimate warming around 2.7°C by the end of the 21st century relative to 1850-1900.	2.1°C–3.5°C
SSP3–7.0	SSP3–7.0 is a medium to high reference scenario resulting from no additional climate policy under the SSP3 socioeconomic development narrative. SSP3-7.0 has particularly high non-CO_2 emissions, including high aerosols emissions	2.8°C–4.6°C
SSP5–8.5	SSP5–8.5 is a high reference scenario with no additional climate policy.	3.3°C–5.7°C

SI Table 2. Image dataset used to determine mean (SD/SE) framework porosity values (replicates for each class varied depending on image availability and quality). Images provided by: Chris Perry; Lauren Toth; Paul Blanchon; Aaron O'Dea; Erin Dillon; Dan Muhs; Ken Johnson; William Precht.

Image code	Location source	Framework components (%)	Stacking porosity (%)
***Acropora palmata* dominated**			
PFF-Ap-1	Pleistocene - Falmouth Fm, Jamaica	64.86	35.14
PFF-Ap-2	Pleistocene - Falmouth Fm, Jamaica	61.77	38.23
PBO-Ap-1	Pleistocene - Bonaire	61.41	38.59
HMB-Ap-1	Holocene - Miami Beach, Florida, USA	62.71	37.29
HMB-Ap-2	Holocene - Miami Beach, Florida, USA	66.02	33.98
PXC-Ap-1	Pleistocene - Xcaret, Mexico	57.15	42.85
PXC-Ap-2	Pleistocene - Xcaret, Mexico	58.27	41.73
PHB-Ap-1	Pleistocene - HoleTown, Barbados	63.29	36.71
PHB-Ap-2	Pleistocene - HoleTown, Barbados	63.00	37.00
PHB-Ap-3	Pleistocene - HoleTown, Barbados	63.66	36.34
PRB-Ap-1	Pleistocene - River Bay, Barbados	59.37	40.63
PRB-Ap-2	Pleistocene - River Bay, Barbados	68.11	31.89
PMB-Ap-1	Pleistocene - Middle Bay, Barbados	63.80	36.20
PMB-Ap-2	Pleistocene - Middle Bay, Barbados	62.65	37.35
PMB-Ap-3	Pleistocene - Middle Bay, Barbados	70.13	29.87
	**Mean**	**63.08**	**36.92**
	**Sd**	**3.43**	**3.43**
	**Se**	**0.89**	**0.89**
***Acropora cervicornis* dominated**			
HED-Ac-1	Holocene - Enriquillo Basin, Dominican Republic	41.38	58.62
HED-Ac-2	Holocene - Enriquillo Basin, Dominican Republic	49.60	50.40
HED-Ac-3	Holocene - Enriquillo Basin, Dominican Republic	42.46	57.54
HED-Ac-4	Holocene - Enriquillo Basin, Dominican Republic	50.75	49.25
HED-Ac-5	Holocene - Enriquillo Basin, Dominican Republic	45.18	54.82
HED-Ac-6	Holocene - Enriquillo Basin, Dominican Republic	35.39	64.61
HED-Ac-7	Holocene - Enriquillo Basin, Dominican Republic	44.37	55.63
HSB-Ac-1	Holocene - Sweet Bocas, Panama	44.42	55.58
HSB-Ac-2	Holocene - Sweet Bocas, Panama	45.41	54.59
HSB-Ac-3	Holocene - Sweet Bocas, Panama	43.20	56.80
PGC-Ac-1	Pleistocene - Guantanamo Bay, Cuba	45.69	54.31
PGC-Ac-2	Pleistocene - Guantanamo Bay, Cuba	46.65	53.35
	**Mean**	**44.54**	**55.46**
	**Sd**	**3.94**	**3.94**
	**Se**	**1.14**	**1.14**
**Branching *Porites* spp. dominated**			
PFF-Pp-1	Pleistocene - Falmouth Fm, Jamaica	58.67	41.33
PFF-Pp-2	Pleistocene - Falmouth Fm, Jamaica	62.99	37.01
PFF-Pp-3	Pleistocene - Falmouth Fm, Jamaica	66.58	33.42
PXC-Pp-1	Pleistocene - Xcaret, Mexico	44.12	55.88
HSB-Pp-1	Holocene - Sweet Bocas, Panama	46.79	53.21
HSB-Pp-2	Holocene - Sweet Bocas, Panama	44.62	55.38
HSB-Pp-3	Holocene - Sweet Bocas, Panama	50.36	49.64
MJA-Pp-1	Miocene – north/central Jamaica	47.72	52.28
	**Mean**	**52.73**	**47.27**
	**Sd**	**8.77**	**8.77**
	**Se**	**3.10**	**3.10**

**Submassive and encrusting taxa dominated**			
HED-SubEnc-1	Holocene - Enriquillo Basin, Dominican Republic	80.02	19.98
HED-SubEnc-2	Holocene - Enriquillo Basin, Dominican Republic	64.63	35.37
HED-SubEnc-3	Holocene - Enriquillo Basin, Dominican Republic	74.49	25.51
HED-SubEnc-4	Holocene - Enriquillo Basin, Dominican Republic	65.60	34.40
HED-SubEnc-5	Holocene - Enriquillo Basin, Dominican Republic	58.06	41.94
HED-SubEnc-6	Holocene - Enriquillo Basin, Dominican Republic	71.57	28.43
HED-SubEnc-7	Holocene - Enriquillo Basin, Dominican Republic	69.96	30.04
	**Mean**	**69.19**	**30.81**
	**Sd**	**7.18**	**7.18**
	**Se**	**2.71**	**2.71**
**Submassive and branched taxa dominated**			
HED-SubBr-1	Holocene - Enriquillo Basin, Dominican Republic	66.76	33.24
HED-SubBr-2	Holocene - Enriquillo Basin, Dominican Republic	70.89	29.11
HED-SubBr-3	Holocene - Enriquillo Basin, Dominican Republic	62.84	37.16
HED-SubBr-4	Holocene - Enriquillo Basin, Dominican Republic	57.04	42.96
HED-SubBr-5	Holocene - Enriquillo Basin, Dominican Republic	69.35	30.65
HED-SubBr-6	Holocene - Enriquillo Basin, Dominican Republic	59.81	40.19
HED-SubBr-7	Holocene - Enriquillo Basin, Dominican Republic	70.28	29.72
HED-SubBr-8	Holocene - Enriquillo Basin, Dominican Republic	62.16	37.84
HED-SubBr-9	Holocene - Enriquillo Basin, Dominican Republic	58.66	41.34
PXC-SubBr-10	Pleistocene - Xcaret, Mexico	52.74	47.26
PXC-SubBr-11	Pleistocene - Xcaret, Mexico	53.86	46.14
PGC-SubBr-12	Pleistocene - Guantanamo Bay, Cuba	59.21	40.79
	**Mean**	**61.97**	**38.03**
	**Sd**	**6.22**	**6.22**
	**Se**	**1.80**	**1.80**
***Orbicella* spp. dominated**			
PKF-Orb-1	Pleistocene - Key Largo Limestone, Florida	60.66	39.34
PKF-Orb-2	Pleistocene - Key Largo Limestone, Florida	76.33	23.67
PKF-Orb-3	Pleistocene - Key Largo Limestone, Florida	72.05	27.95
PKF-Orb-4	Pleistocene - Key Largo Limestone, Florida	61.20	38.80
PGC-Orb-5	Pleistocene - Guantanamo Bay, Cuba	78.90	21.10
HSB-Orb-6	Holocene - Sweet Bocas, Panama	61.75	38.25
PWK-Orb-1	Pleistocene - Key Largo Limestone, Florida	57.04	42.96
PWK-Orb-2	Pleistocene - Key Largo Limestone, Florida	64.87	35.13
PWK-Orb-3	Pleistocene - Key Largo Limestone, Florida	59.74	40.26
	**Mean**	**65.84**	**34.16**
	**Sd**	**8.28**	**8.28**
	**Se**	**2.76**	**2.76**
**Head and massive taxa dominated**			
HED-Mass-1	Holocene - Enriquillo Basin, Dominican Republic	76.71	23.29
HED-Mass-2	Holocene - Enriquillo Basin, Dominican Republic	72.03	27.97
HED-Mass-3	Holocene - Enriquillo Basin, Dominican Republic	74.14	25.86
	**Mean**	**74.29**	**25.71**
	**Sd**	**2.34**	**2.34**
	**Se**	**1.35**	**1.35**

SI Table 3. Results of paired t-tests comparing reef accretion potential (RAP_{max}) calculated using both the originally proposed and revised porosity factors; a) within different Western Atlantic sub-regions from Perry et al (2018); b) grouped by coral % cover classes; c) grouped by net carbonate production rate classes; d) for a sub-set of sites in the Mexican Caribbean that differ in overall coral cover and coral assemblage type.

a)

Sub-region	Perry et al. 2018		Revised RAP_{max}				
	**Mean**	**SD**	**Mean**	**SD**	***t (# pairs)***	***p***	**95% CI**
All countries	1.9	2.2	1.6	1.8	4.083 (96)	0.0001	-0.346, -0.119
Florida & Gt Antilles	0.4	0.8	0.3	0.8	0.811 (12)	0.4342	-0.061, 0.028
Mesoamerican reef	0.6	1.4	0.5	1.3	2.266 (17)	0.0377	-0.239, -0.007
Northern Lesser Antilles	1.5	1.1	1.5	1.1	1.121 (27)	0.2727	-0.037, 0.011
Southern Lesser Antilles	2.2	1.9	2.1	1.8	0.882 (26)	0.3859	-0.131, 0.052
Leeward Antilles	4.9	2.7	3.5	1.9	6.613 (13)	0.0001	-1.771, -0.898

b)

Coral cover (%)	Perry et al. 2018		Revised RAP_{max}				
	**Mean**	**SD**	**Mean**	**SD**	***t (# pairs)***	***p***	**95% CI**
<10%	0.1	0.6	0.1	0.6	0.264 (18)	0.7946	-0.019, 0.024
10.0-19.9%	1.1	1.1	1.0	1.1	2.181 (40)	0.0353	-0.065, -0.002
20.0 – 39.9%	2.6	1.8	2.5	1.6	1.486 (26)	0.1499	-0.330, 0.053
>40.0%	5.6	1.9	4.3	1.2	5.145 (12)	0.0003	-1.891, -0.757

c)

Carbonate budget (G, kg $CaCO_3$ m^{-2} yr^{-1})	Perry et al. 2018		Revised RAP_{max}				
	**Mean**	**SD**	**Mean**	**SD**	***t (# pairs)***	***p***	**95% CI**
<0.00 G	-0.3	0.5	-0.3	0.5	0.377 (20)	0.7101	-0.013, 0.019
0.00-3.99 G	1.2	0.6	1.1	0.5	2.562 (47)	0.0137	-0.112, -0.013
4.00 – 7.99 G	3.4	0.9	3.1	0.7	2.859 (24)	0.0089	-0.593, -0.095
>8.00 G	7.4	1.5	6.3	2.7	4.873 (7)	0.0028	-2.427, -0.804

d)

Site	Perry et al. 2018		Revised RAP_{max}				
	**Mean**	**SD**	**Mean**	**SD**	***t (# pairs)***	***p***	**95% CI**
Limones	17.4	9.4	8.3	4.5	4.516 (6)	0.0063	-14.270, -3.918
Manchones Norte	13.9	9.5	6.7	4.6	3.594 (6)	0.0156	-12.350, -2.051
Nizuc C3	7.3	3.8	3.5	1.8	4.659 (6)	0.0055	-5.960, -1.722
Cayo Arenas 6	1.7	0.9	1.2	1.0	3.021 (6)	0.0294	0.016, 0.131
Radio Pirata	0.5	0.8	0.4	0.4	0.976 (6)	0.3735	-0.607, 0.272
Palancar Jardines	1.4	0.9	1.0	0.3	3.488 (6)	0.0175	-0.550, -0.083
La Catedral	-0.4	0.1	-0.4	0.1		No change	

SI Table 4. Mean percentage changes in a) coral cover, b) coral calcification, c) crustose coralline algae calcification and d) bioerosion for each sector of each sub-region under different Shared Socioeconomic Pathway (SSP) scenarios at 2040, 2060 and 2100. Changes are % declines compared to preceding reference date (for 2040 this is relative to the date of site surveying).

a) Total projected % changes in live coral cover

	SSP1-2.6			SSP2-4.5			SSP3-7.0			SSP5-8.5		
	Census -2040	2041-2060	2061-2100	Census -2040	2041-2060	2061-2100	Census -2040	2041-2060	2061-2100	Census -2040	2041-2060	2061-2100
**Florida**												
Biscayne	-61.0	-58.4	-82.7	-59.1	-62.9	-99.6	-59.7	-68.4	-100.0	-63.2	-86.1	-100.0
Upper Keys	-62.6	-58.4	-82.7	-58.5	-63.6	-99.7	-58.0	-68.4	-100.0	-63.0	-86.2	-100.0
Mid-Upper Transition	-62.4	-58.4	-82.7	-62.5	-60.3	-99.6	-55.6	-67.0	-99.9	-63.2	-83.8	-100.0
Middle Keys	-63.6	-58.4	-82.7	-62.0	-58.9	-99.5	-58.2	-67.0	-99.9	-62.9	-83.2	-100.0
Lower Keys	-61.6	-58.4	-82.7	-63.8	-59.9	-99.5	-57.9	-67.0	-99.9	-63.0	-84.8	-100.0
**Mexico & Gulf of Mexico**												
Veracruz	-56.5	-58.4	-82.7	-56.5	-58.4	-99.6	-50.6	-68.5	-100.0	-56.5	-85.9	-100.0
Banco de Campeche	-55.2	-58.4	-82.7	-55.8	-58.4	-97.3	-31.9	-59.2	-99.9	-54.4	-73.7	-100.0
Northern Quintana Roo	-61.3	-58.4	-82.7	-60.1	-58.4	-99.5	-48.2	-64.1	-99.9	-61.0	-84.2	-100.0
Cozumel	-61.1	-58.4	-82.7	-60.2	-58.4	-99.5	-51.1	-63.8	-99.9	-62.3	-86.7	-100.0
Central Quintana Roo	-61.9	-58.4	-82.7	-61.9	-58.4	-99.5	-55.4	-63.8	-99.9	-60.2	-80.1	-100.0
Banco Chinchorro	-60.2	-58.4	-82.7	-60.2	-58.4	-99.1	-54.9	-62.9	-99.9	-58.4	-77.7	-100.0
South Quintana Roo	-61.9	-58.4	-82.7	-60.2	-58.4	-99.0	-57.0	-62.1	-99.9	-61.9	-76.2	-100.0
**Bonaire**												
North Exposed	-58.4	-58.4	-82.7	-63.5	-58.4	-96.0	-45.9	-58.4	-99.8	-61.9	-67.0	-100.0
North	-58.4	-58.4	-82.7	-63.5	-58.4	-96.0	-45.9	-58.4	-99.8	-61.9	-66.0	-100.0
Central	-58.4	-58.4	-82.7	-63.5	-58.4	-96.0	-45.9	-58.4	-99.8	-61.9	-67.0	-100.0
Klein Bonaire	-58.4	-58.4	-82.7	-63.5	-58.4	-96.0	-45.9	-58.4	-99.8	-61.9	-67.0	-100.0
South	-58.4	-58.4	-82.7	-63.5	-58.4	-96.0	-45.9	-58.4	-99.8	-61.9	-67.0	-100.0

b) Projected % changes in coral calcification rates from interactive effects of sea-surface temperature and ocean acidification

	SSP1-2.6			SSP2-4.5			SSP3-7.0			SSP5-8.5		
	Census -2040	2041-2060	2061-2100	Census -2040	2041-2060	2061-2100	Census -2040	2041-2060	2061-2100	Census -2040	2041-2060	2061-2100
**Florida**												
Biscayne	-2.6	-4.7	-4.8	-3.0	-7.1	-15.2	-3.1	-8.9	-32.5	-3.4	-11.2	-51.6
Upper Keys	-2.8	-4.9	-5.1	-3.2	-7.5	-16.0	-3.2	-9.4	-34.0	-3.4	-11.7	-53.8
Mid-Upper Transition	-2.8	-4.9	-5.1	-3.1	-7.4	-15.8	-3.1	-9.3	-33.7	-3.5	-11.7	-53.9
Middle Keys	-2.7	-4.9	-5.1	-3.1	-7.3	-15.6	-3.1	-9.2	-33.6	-3.4	-11.6	-53.7
Lower Keys	-2.7	-4.8	-4.9	-3.0	-7.1	-15.2	-3.1	-9.0	-32.8	-3.4	-11.4	-52.2
**Mexico & Gulf of Mexico**												
Veracruz	-2.8	-4.9	-5.1	-2.9	-6.9	-15.4	-3.1	-9.1	-32.9	-3.4	-11.3	-53.0
Banco de Campeche	-2.7	-4.6	-4.8	-2.8	-6.7	-14.5	-3.0	-8.8	-31.4	-3.2	-10.6	-49.0
Northern Quintana Roo	-2.6	-4.5	-4.7	-2.8	-6.7	-14.5	-3.0	-8.7	-31.2	-3.2	-10.6	-49.2
Cozumel	-2.6	-4.6	-4.7	-2.8	-6.7	-14.5	-3.0	-8.7	-31.2	-3.2	-10.7	-49.3
Central Quintana Roo	-2.6	-4.5	-4.6	-2.8	-6.6	-14.4	-2.9	-8.6	-30.9	-3.2	-10.5	-48.9
Banco Chinchorro	-2.7	-4.6	-4.7	-2.8	-6.8	-14.6	-3.0	-8.7	-31.1	-3.2	-10.7	-49.2
South Quintana Roo	-2.7	-4.6	-4.8	-2.9	-6.8	-14.7	-3.0	-8.7	-30.9	-3.3	-10.7	-49.0
**Bonaire**												
North Exposed	-2.5	-4.5	-4.7	-2.7	-6.2	-13.8	-2.8	-7.8	-28.6	-3.1	-9.6	-45.9
North	-2.5	-4.5	-4.7	-2.7	-6.2	-13.8	-2.8	-7.8	-28.6	-3.1	-9.6	-45.9
Central	-2.5	-4.5	-4.7	-2.7	-6.2	-13.8	-2.8	-7.8	-28.6	-3.1	-9.6	-45.9
Klein Bonaire	-2.5	-4.5	-4.7	-2.7	-6.2	-13.8	-2.8	-7.8	-28.6	-3.1	-9.6	-45.9
South	-2.5	-4.5	-4.7	-2.7	-6.2	-13.8	-2.8	-7.8	-28.6	-3.1	-9.6	-45.9

c) Projected % changes in coralline algae calcification rates from interactive effects of sea-surface temperature and ocean acidification

	SSP1-2.6			SSP2-4.5			SSP3-7.0			SSP5-8.5		
	Census -2040	2041-2060	2061-2100	Census -2040	2041-2060	2061-2100	Census -2040	2041-2060	2061-2100	Census -2040	2041-2060	2061-2100
**Florida**												
Biscayne	-3.9	-5.7	-5.2	-4.2	-7.7	-11.8	-4.6	-9.4	-18.5	-4.6	-10.2	-23.9
Upper Keys	-4.0	-5.8	-5.3	-4.3	-8.0	-12.2	-4.6	-9.7	-19.1	-4.7	-10.5	-24.7
Mid-Upper Transition	-4.0	-5.8	-5.4	-4.4	-8.0	-12.3	-4.6	-9.7	-19.2	-4.7	-10.5	-24.7
Middle Keys	-4.0	-5.8	-5.3	-4.4	-8.0	-12.2	-4.7	-9.7	-19.1	-4.7	-10.5	-24.5
Lower Keys	-3.9	-5.7	-5.1	-4.3	-7.7	-11.8	-4.6	-9.4	-18.6	-4.7	-10.3	-24.0
**Mexico & Gulf of Mexico**												
Veracruz	-4.0	-5.9	-5.4	-4.3	-7.8	-12.3	-4.7	-9.6	-18.8	-4.7	-10.3	-24.2
Banco de Campeche	-4.0	-5.8	-5.4	-4.3	-7.8	-12.3	-4.6	-9.5	-19.1	-4.7	-10.4	-24.7
Northern Quintana Roo	-3.9	-5.6	-5.1	-4.2	-7.7	-12.0	-4.6	-9.4	-18.8	-4.6	-10.3	-24.4
Cozumel	-3.9	-5.7	-5.1	-4.3	-7.7	-11.9	-4.7	-9.4	-18.7	-4.7	-10.3	-24.3
Central Quintana Roo	-3.9	-5.6	-5.1	-4.2	-7.7	-11.9	-4.6	-9.4	-18.8	-4.6	-10.2	-24.4
Banco Chinchorro	-4.0	-5.8	-5.2	-4.3	-7.8	-12.1	-4.7	-9.6	-18.9	-4.8	-10.4	-24.5
South Quintana Roo	-4.0	-5.8	-5.3	-4.4	-7.9	-12.1	-4.8	-9.6	-19.1	-4.8	-10.5	-24.7
**Bonaire**												
North Exposed	-4.0	-5.8	-5.5	-4.3	-7.7	-12.2	-4.7	-9.4	-19.2	-4.6	-10.3	-24.7
North	-3.9	-5.8	-5.5	-4.3	-7.7	-12.2	-4.8	-9.4	-19.2	-4.6	-10.3	-24.7
Central	-3.9	-5.8	-5.5	-4.3	-7.7	-12.2	-4.7	-9.4	-19.2	-4.6	-10.3	-24.7
Klein Bonaire	-3.9	-5.8	-5.5	-4.3	-7.7	-12.2	-4.7	-9.4	-19.2	-4.6	-10.3	-24.7
South	-3.9	-5.8	-5.5	-4.3	-7.7	-12.2	-4.7	-9.4	-19.2	-4.6	-10.3	-24.7

d) Projected % changes in bioerosion rates from interactive effects of sea-surface temperature and ocean acidification

	SSP1-2.6			SSP2-4.5			SSP3-7.0			SSP5-8.5		
	Census -2040	2041-2060	2061-2100	Census -2040	2041-2060	2061-2100	Census -2040	2041-2060	2061-2100	Census -2040	2041-2060	2061-2100
**Florida**												
Biscayne	2.2	4.5	7.4	2.8	5.6	15.2	1.3	5.2	33.1	2.7	9.5	54.6
Upper Keys	2.6	5.2	8.0	3.4	6.1	16.0	2.2	5.9	34.5	3.0	10.1	56.5
Mid-Upper Transition	2.6	5.1	8.0	2.6	5.4	15.0	1.5	5.2	33.6	3.0	10.0	56.4
Middle Keys	2.4	4.9	7.8	2.4	5.2	14.8	1.1	5.2	33.5	2.8	9.8	56.2
Lower Keys	2.6	4.9	8.0	2.8	5.8	15.3	1.3	5.6	33.7	2.9	9.8	55.7
**Mexico & Gulf of Mexico**												
Veracruz	2.7	4.8	7.8	1.9	4.5	13.9	1.1	5.0	33.1	2.7	9.5	56.6
Banco de Campeche	2.0	3.6	6.3	1.5	3.7	11.2	1.3	4.3	28.6	1.8	6.9	46.1
Northern Quintana Roo	2.1	4.0	7.0	1.6	4.0	12.6	1.1	4.5	29.2	1.8	7.3	47.7
Cozumel	2.2	4.0	7.1	1.6	4.0	12.8	0.9	4.6	29.6	1.8	7.4	48.5
Central Quintana Roo	2.0	4.0	7.0	1.5	3.9	12.5	0.8	4.2	28.5	1.8	7.3	47.4
Banco Chinchorro	2.0	3.8	6.6	1.4	3.7	12.4	0.5	3.8	28.0	1.6	7.0	47.1
South Quintana Roo	1.8	3.6	6.4	1.3	3.5	12.2	0.2	3.4	27.3	1.5	6.7	46.2
**Bonaire**												
North Exposed	1.5	3.0	5.2	0.9	2.2	9.6	-0.5	1.3	21.3	1.2	4.0	39.4
North	1.5	3.1	5.3	0.9	2.2	9.5	-0.6	1.3	21.3	1.3	4.0	39.4
Central	1.5	3.1	5.3	0.9	2.2	9.6	-0.6	1.3	21.3	1.3	4.0	39.4
Klein Bonaire	1.5	3.1	5.3	0.9	2.2	9.6	-0.6	1.3	21.3	1.3	4.0	39.4
South	1.5	3.1	5.3	0.9	2.2	9.6	-0.6	1.3	21.3	1.3	4.0	39.5

SI Table 5. Climate projection models used for estimating sea-surface temperature under each Shared Socioeconomic Pathway (SSP) scenarios

	Model name	historical	SSP1–2.6	SSP2–4.5	SSP3–7.0	SSP5–8.5
1	ACCESS-CM2	X	X	X	X	X
2	ACCESS-ESM1-5	X	X	X	X	X
3	AWI-CM-1-1-MR	X	X	X	X	X
4	BCC-CSM2-MR	X	X	X	X	X
5	CanESM5	X	X	X	X	X
6	CESM2-WACCM	X	X	X	X	X
7	CMCC-CM2-SR5	X	X	X	X	X
8	CNRM-CM6-1	X	X	X	X	X
9	CNRM-CM6-1-HR	X	X	X	X	X
10	CNRM-ESM2-1	X	X	X	X	X
11	EC-EARTH3	X	X	X	X	X
12	EC-EARTH3-Veg	X	X	X	X	X
13	GFDL-CM4	X		X		X
14	GFDL-ESM4	X	X	X	X	X
15	HADGEM3-GC31-LL	X	X	X		X
16	HadGEM3-GC31-MM	X	X			X
17	IPSL-CM6A-LR	X	X	X	X	X
18	MPI-ESM1-2-HR	X	X	X	X	X
19	MPI-ESM1-2-LR	X	X	X	X	X
20	NESM3	X	X	X		X
21	NorESM2-LM	X	X	X	X	X
22	NorESM2-MM	X	X	X	X	X
23	UKESM1-0-LL	X	X	X	X	X

SI Table 6. Climate projection models used for estimating pH under each Shared Socioeconomic Pathway (SSP) scenarios

	model_name	historical	SSP1–2.6	SSP2–4.5	SSP3–7.0	SSP5–8.5
1	CESM2	X	X	X	X	X
2	CESM2-WACCM	X	X	X	X	X
3	CNRM-ESM2-1	X	X	X	X	X
4	GFDL-ESM4	X	X	X	X	X
5	IPSL-CM6A-LR	X	X	X	X	X
6	MIROC-ES2L	X	X	X	X	X
7	MPI-ESM1-2-HR	X	X	X	X	X
8	MPI-ESM1-2-LR	X	X	X	X	X
9	NorESM2-LM	X	X	X	X	X
10	NorESM2-MM	X	X	X	X	X
11	UKESM1-0-LL	X	X	X	X	X

SI Table 7. Total projected sea-level rise (SLR) (m) and rates of sea-level rise (mm yr^{-1}) under different Shared Socioeconomic Pathway (SSP) scenarios and for different time points through to 2100 for different areas analysed in this study. Data from the IPCC 6th Assessment Report (AR6) (Fox-Kemper et al. 2021) accessed via the NASA Sea Level Projection Tool (Garner et al. 2021) – see below for full citation information.

	**Total SLR (m)**						**Projected rate of SLR (mm yr^{-1})**				
**Location**	**2040 (SSP1-19)**	**2040 (SSP1-26)**	**2040 (SSP2-45)**	**2040 (SSP3-70)**	**2040 (SSP5-8.5)**		**2040 (SSP1-19)**	**2040 (SSP1-26)**	**2040 (SSP2-45)**	**2040 (SSP3-70)**	**2040 (SSP5-8.5)**
Florida - Upper Keys/Biscayne	0.19	0.20	0.21	0.21	0.22		6.0	6.0	7.0	7.0	8.0
Florida - Middle Keys	0.21	0.21	0.22	0.22	0.23		6.6	6.6	7.2	7.2	8.1
Florida - Lower Keys	0.20	0.21	0.21	0.21	0.22		6.2	6.3	7.0	7.0	7.8
Mexico - Veracruz area	0.18	0.19	0.19	0.19	0.20		5.0	5.0	6.0	6.0	7.0
Mexico - Campeche Bank	0.24	0.25	0.25	0.25	0.26		7.0	7.0	8.0	8.0	8.0
Mexico - Cancun to Sian Ka'an	0.22	0.23	0.23	0.23	0.24		7.0	7.0	7.0	7.0	8.0
Mexico - Sian Ka'an to Mahuhual	0.24	0.25	0.26	0.26	0.27		7.0	7.0	8.0	8.0	9.0
Bonaire	0.17	0.17	0.18	0.18	0.19		5.0	5.0	6.0	6.0	6.0
	**2060 (SSP1-19)**	**2060 (SSP1-26)**	**2060 (SSP2-45)**	**2060 (SSP3-70)**	**2060 (SSP5-8.5)**		**2060 (SSP1-19)**	**2060 (SSP1-26)**	**2060 (SSP2-45)**	**2060 (SSP3-70)**	**2060 (SSP5-8.5)**
Florida - Upper Keys/Biscayne	0.38	0.40	0.45	0.47	0.51		5.0	6.0	8.0	9.0	9.0
Florida - Middle Keys	0.39	0.42	0.46	0.49	0.53		5.7	6.5	8.1	9.0	9.9
Florida - Lower Keys	0.38	0.40	0.45	0.48	0.51		5.0	6.0	7.0	8.0	9.0
Mexico - Veracruz area	0.31	0.36	0.41	0.43	0.47		4.0	5.0	7.0	8.0	8.0
Mexico - Campeche Bank	0.46	0.48	0.52	0.55	0.59		6.0	7.0	8.0	9.0	10.0
Mexico - Cancun to Sian Ka'an	0.43	0.45	0.50	0.53	0.56		6.0	7.0	8.0	9.0	10.0
Mexico - Sian Ka'an to Mahuhual	0.47	0.50	0.54	0.57	0.61		7.0	8.0	9.0	10.0	11.0
Bonaire	0.33	0.35	0.40	0.42	0.46		5.0	5.0	7.0	8.0	9.0
	**2100 (SSP1-19)**	**2100 (SSP1-26)**	**2100 (SSP2-45)**	**2100 (SSP3-70)**	**2100 (SSP5-8.5)**		**2100 (SSP1-19)**	**2100 (SSP1-26)**	**2100 (SSP2-45)**	**2100 (SSP3-70)**	**2100 (SSP5-8.5)**
Florida - Upper Keys/Biscayne	0.54	0.59	0.71	0.82	0.91		6.0	6.0	9.0	12.0	14.0
Florida - Middle Keys	0.55	0.60	0.72	0.84	0.93		5.8	6.2	9.2	12.3	14.0
Florida - Lower Keys	0.53	0.58	0.71	0.83	0.92		5.8	6.1	9.0	12.2	13.9
Mexico - Veracruz area	0.64	0.53	0.64	0.77	0.86		4.0	5.0	8.0	11.0	13.0
Mexico - Campeche Bank	0.47	0.69	0.81	0.93	1.02		6.0	7.0	9.0	13.0	15.0
Mexico - Cancun to Sian Ka'an	0.61	0.66	0.79	0.91	1.00		6.0	6.0	9.0	13.0	15.0
Mexico - Sian Ka'an to Mahuhual	0.67	0.72	0.85	0.97	1.06		7.0	7.0	10.0	13.0	15.0
Bonaire	0.47	0.51	0.64	0.76	0.85		4.0	5.0	8.0	11.0	13.0

Sea-level rise projections based on chapter 9 of Working Group 1 contribution to the IPCC Sixth Assessment Report, the Framework for Assessment of Changes To Sea-level (FACTS): Fox-Kemper, B., H.T. Hewitt, C. Xiao, G. Aðalgeirsdóttir, S.S. Drijfhout, T.L. Edwards, N.R. Golledge, M. Hemer, R.E. Kopp, G. Krinner, A. Mix, D. Notz, S. Nowicki, I.S. Nurhati, L. Ruiz, J.-B. Sallée, A.B.A. Slangen, and Y. Yu, 2021: Ocean, Cryosphere and Sea Level Change. In Climate Change 2021: The Physical Science Basis. Contribution of Working Group I to the Sixth Assessment Report of the Intergovernmental Panel on Climate Change [Masson-Delmotte, V., P. Zhai, A. Pirani, S.L. Connors, C. Péan, S. Berger, N. Caud, Y. Chen, L. Goldfarb, M.I. Gomis, M. Huang, K. Leitzell, E. Lonnoy, J.B.R. Matthews, T.K. Maycock, T. Waterfield, O. Yelekçi, R. Yu, and B. Zhou (eds.)]. Cambridge University Press, Cambridge, United Kingdom and New York, NY, USA, pp. 1211–1362, doi:10.1017/9781009157896.011.

Data extracted from the NASA Sea Level Projection Tool: Garner, G. G., T. Hermans, R. E. Kopp, A. B. A. Slangen, T. L. Edwards, A. Levermann, S. Nowikci, M. D. Palmer, C. Smith, B. Fox-Kemper, H. T. Hewitt, C. Xiao, G. Aðalgeirsdóttir, S. S. Drijfhout, T. L. Edwards, N. R. Golledge, M. Hemer, R. E. Kopp, G. Krinner, A. Mix, D. Notz, S. Nowicki, I. S. Nurhati, L. Ruiz, J-B. Sallée, Y. Yu, L. Hua, T. Palmer, B. Pearson, 2021. IPCC AR6 Sea-Level Rise Projections. Version 20210809. PO.DAAC, CA, USA. Dataset accessed [2024-02-06] at https://podaac.jpl.nasa.gov/announcements/2021-08-09-Sea-level-projections-from-the-IPCC-6th-Assessment-Report.

SI Table 8. Survey sites by country and sub-region and calculated present and projected future reef accretion potential (RAP_{max}) rates (mm yr^{-1})

						RAP_{max} rate (mm yr^{-1})												
Survey year	Sector	Site name or code	Depth (m)	Coral cover (%)	Net G	Present	2040				2060				2100			
							SSP 1-2.6	SSP 2-4.5	SSP 3-7.0	SSP 5-8.5	SSP 1-2.6:	SSP 2-4.5	SSP 3-7.0	SSP 5-8.5	SSP 1-2.6:	SSP 2-4.5	SSP 3-7.0	SSP 5-8.5
Florida Keys																		
2018	Biscayne	1669	3	1	-0.66	-0.35	-0.38	-0.38	-0.38	-0.38	-0.38	-0.38	-0.39	-0.40	-0.39	-0.41	-0.44	-0.47
2018	Biscayne	1101	3	13	0.25	0.15	-0.09	-0.08	-0.09	-0.09	-0.08	-0.10	-0.12	-0.16	-0.16	-0.20	-0.22	-0.25
2018	Biscayne	1168	3	3	-1.00	-0.44	-0.46	-0.46	-0.46	-0.46	-0.46	-0.47	-0.47	-0.47	-0.47	-0.48	-0.50	-0.52
2016	Biscayne	1049	3	20	0.52	0.33	-0.07	-0.06	-0.06	-0.09	-0.06	-0.08	-0.11	-0.18	-0.18	-0.25	-0.28	-0.31
2018	Biscayne	1306	4	0	-1.12	-0.31	-0.32	-0.32	-0.32	-0.32	-0.32	-0.32	-0.32	-0.32	-0.32	-0.33	-0.34	-0.36
2018	Biscayne	1085	4	10	0.39	0.18	0.00	0.00	0.00	-0.01	0.00	-0.01	-0.03	-0.07	-0.06	-0.10	-0.12	-0.13
2018	Biscayne	1519	4	5	-0.14	-0.04	-0.13	-0.13	-0.13	-0.13	-0.13	-0.13	-0.14	-0.16	-0.16	-0.18	-0.20	-0.21
2018	Biscayne	1091	4	7	0.13	0.06	-0.05	-0.05	-0.05	-0.06	-0.05	-0.06	-0.07	-0.10	-0.09	-0.12	-0.14	-0.16
2018	Biscayne	1095	4	13	-0.10	0.00	-0.18	-0.18	-0.18	-0.19	-0.18	-0.19	-0.21	-0.25	-0.24	-0.28	-0.30	-0.33
2016	Biscayne	1012	5	15	0.62	0.32	-0.04	-0.04	-0.03	-0.06	-0.03	-0.05	-0.08	-0.15	-0.14	-0.21	-0.23	-0.25
2018	Biscayne	1363	6	15	-0.07	0.02	-0.13	-0.13	-0.13	-0.14	-0.13	-0.15	-0.15	-0.18	-0.18	-0.21	-0.22	-0.23
2018	Biscayne	1097	7	15	0.09	0.10	-0.17	-0.16	-0.17	-0.18	-0.17	-0.18	-0.20	-0.26	-0.25	-0.30	-0.33	-0.35
2018	Biscayne	1130	7	16	-0.36	-0.09	-0.23	-0.23	-0.23	-0.24	-0.23	-0.25	-0.25	-0.28	-0.28	-0.31	-0.32	-0.33
2018	Biscayne	1226	7	3	-0.38	-0.13	-0.20	-0.20	-0.20	-0.20	-0.20	-0.21	-0.21	-0.23	-0.22	-0.24	-0.26	-0.28
2018	Biscayne	1100	7	3	-0.18	-0.10	-0.11	-0.11	-0.11	-0.11	-0.11	-0.11	-0.12	-0.12	-0.12	-0.13	-0.15	-0.17
2018	Biscayne	1086	8	8	0.03	0.02	-0.10	-0.10	-0.10	-0.11	-0.10	-0.11	-0.11	-0.14	-0.14	-0.17	-0.19	-0.21
2016	Biscayne	1039	8	5	0.05	0.03	-0.08	-0.08	-0.08	-0.09	-0.08	-0.10	-0.10	-0.13	-0.12	-0.15	-0.17	-0.20
2018	Biscayne	1080	8	1	-0.09	-0.06	-0.06	-0.06	-0.06	-0.06	-0.06	-0.06	-0.06	-0.07	-0.06	-0.07	-0.08	-0.10
2018	Biscayne	1630	9	0	-0.09	-0.03	-0.03	-0.03	-0.03	-0.03	-0.03	-0.03	-0.03	-0.03	-0.03	-0.03	-0.03	-0.03
2018	Biscayne	1082	9	1	-1.78	-0.91	-0.92	-0.92	-0.92	-0.92	-0.92	-0.93	-0.93	-0.93	-0.93	-0.94	-0.97	-1.00
2016	Biscayne	1123	9	5	-0.43	-0.19	-0.25	-0.25	-0.25	-0.26	-0.26	-0.26	-0.27	-0.28	-0.28	-0.30	-0.32	-0.35
2016	Biscayne	1056	11	1	-0.16	-0.09	-0.11	-0.11	-0.11	-0.12	-0.12	-0.12	-0.12	-0.13	-0.12	-0.14	-0.15	-0.17
2018	Biscayne	1230	12	6	-0.13	-0.03	-0.13	-0.13	-0.13	-0.14	-0.13	-0.14	-0.15	-0.17	-0.17	-0.19	-0.21	-0.23
2018	Upper Keys	1254	2	0	-0.24	-0.08	-0.09	-0.09	-0.08	-0.09	-0.09	-0.09	-0.09	-0.09	-0.09	-0.10	-0.11	-0.13

2018	Upper Keys	1067	3	4	0.16	0.08	-0.03	-0.03	-0.03	-0.03	-0.03	-0.04	-0.04	-0.07	-0.06	-0.09	-0.10	-0.12
2018	Upper Keys	1068	3	11	0.40	0.20	-0.03	-0.02	-0.02	-0.04	-0.03	-0.05	-0.06	-0.11	-0.10	-0.14	-0.16	-0.18
2018	Upper Keys	1123	3	2	-0.08	-0.05	-0.07	-0.07	-0.07	-0.07	-0.07	-0.08	-0.08	-0.09	-0.08	-0.09	-0.11	-0.13
2018	Upper Keys	1158	3	0	-0.22	-0.07	-0.07	-0.07	-0.07	-0.07	-0.07	-0.07	-0.07	-0.08	-0.07	-0.08	-0.11	-0.13
2018	Upper Keys	1162	3	0	-0.20	-0.07	-0.07	-0.07	-0.07	-0.07	-0.08	-0.08	-0.08	-0.08	-0.08	-0.08	-0.10	-0.12
2016	Upper Keys	1171	4	2	-0.87	-0.39	-0.41	-0.41	-0.41	-0.41	-0.41	-0.41	-0.41	-0.42	-0.42	-0.43	-0.46	-0.48
2016	Upper Keys	1164	4	2	-0.97	-0.51	-0.53	-0.53	-0.53	-0.53	-0.53	-0.53	-0.53	-0.54	-0.54	-0.55	-0.58	-0.62
2016	Upper Keys	1184	4	5	-1.90	-0.80	-0.88	-0.88	-0.88	-0.88	-0.88	-0.89	-0.89	-0.91	-0.91	-0.93	-0.96	-0.99
2018	Upper Keys	1065	4	22	0.99	0.47	0.05	0.07	0.08	0.04	0.06	0.03	0.01	-0.08	-0.07	-0.14	-0.16	-0.19
2018	Upper Keys	1127	4	4	-0.87	-0.31	-0.36	-0.36	-0.36	-0.36	-0.36	-0.37	-0.37	-0.38	-0.38	-0.40	-0.42	-0.44
2016	Upper Keys	1201	6	31	2.41	1.16	0.17	0.24	0.24	0.17	0.23	0.16	0.11	-0.07	-0.06	-0.22	-0.24	-0.27
2018	Upper Keys	1198	6	2	-0.79	-0.36	-0.37	-0.37	-0.37	-0.37	-0.37	-0.37	-0.37	-0.38	-0.38	-0.39	-0.41	-0.43
2018	Upper Keys	1629	6	1	-1.39	-0.71	-0.72	-0.72	-0.72	-0.72	-0.72	-0.72	-0.72	-0.73	-0.73	-0.74	-0.76	-0.79
2016	Upper Keys	1229	6	3	-0.81	-0.36	-0.39	-0.39	-0.39	-0.39	-0.39	-0.40	-0.40	-0.41	-0.41	-0.42	-0.45	-0.48
2016	Upper Keys	1213	9	3	-1.55	-0.55	-0.60	-0.60	-0.60	-0.60	-0.60	-0.60	-0.61	-0.62	-0.62	-0.63	-0.65	-0.67
2018	Upper Keys	1138	11	3	-0.28	-0.10	-0.16	-0.16	-0.15	-0.16	-0.16	-0.16	-0.17	-0.18	-0.18	-0.19	-0.21	-0.22
2016	Mid-Upper Keys Transition	1247	3	10	-0.11	0.04	-0.25	-0.25	-0.22	-0.25	-0.23	-0.24	-0.27	-0.32	-0.32	-0.37	-0.39	-0.41
2016	Mid-Upper Keys Transition	1257	3	34	2.61	1.45	0.20	0.20	0.30	0.17	0.22	0.18	0.08	-0.15	-0.17	-0.37	-0.40	-0.43
2018	Mid-Upper Keys Transition	1014	3	1	-0.21	-0.10	-0.12	-0.12	-0.12	-0.12	-0.12	-0.12	-0.12	-0.13	-0.13	-0.14	-0.16	-0.19
2018	Mid-Upper Keys Transition	1059	4	11	0.53	0.25	0.05	0.05	0.07	0.04	0.05	0.04	0.03	-0.01	-0.01	-0.04	-0.05	-0.06
2016	Mid-Upper Keys Transition	1294	4	0	-0.24	-0.08	-0.09	-0.09	-0.09	-0.09	-0.09	-0.09	-0.09	-0.09	-0.09	-0.10	-0.11	-0.13
2016	Mid-Upper Keys Transition	1256	4	10	-2.39	-0.79	-1.00	-1.00	-0.98	-1.00	-0.99	-0.99	-1.01	-1.05	-1.05	-1.09	-1.11	-1.14
2018	Mid-Upper Keys Transition	1113	5	2	-0.13	-0.08	-0.10	-0.10	-0.09	-0.10	-0.10	-0.10	-0.10	-0.11	-0.10	-0.11	-0.13	-0.15
2016	Mid-Upper Keys Transition	1265	5	4	-0.66	-0.23	-0.31	-0.31	-0.30	-0.32	-0.31	-0.32	-0.32	-0.34	-0.34	-0.36	-0.38	-0.41
2016	Mid-Upper Keys Transition	1326	6	2	-2.24	-0.96	-0.99	-0.99	-0.98	-0.99	-0.99	-0.99	-0.99	-1.01	-1.00	-1.02	-1.04	-1.07
2016	Mid-Upper Keys Transition	1296	6	1	-0.87	-0.32	-0.34	-0.34	-0.33	-0.34	-0.34	-0.34	-0.34	-0.35	-0.35	-0.35	-0.37	-0.39
2016	Mid-Upper Keys Transition	1282	6	9	-0.86	-0.05	-1.05	-1.05	-0.95	-1.05	-0.99	-1.02	-1.10	-1.27	-1.29	-1.45	-1.48	-1.51
2016	Mid-Upper Keys Transition	1312	6	1	-0.80	-0.29	-0.31	-0.31	-0.31	-0.31	-0.32	-0.32	-0.32	-0.32	-0.32	-0.33	-0.35	-0.37
2016	Mid-Upper Keys Transition	1284	7	4	-0.30	-0.11	-0.11	-0.11	-0.11	-0.12	-0.12	-0.12	-0.12	-0.13	-0.12	-0.14	-0.18	-0.22

2018	Mid-Upper Keys Transition	1155	7	0	-0.11	-0.04	-0.04	-0.04	-0.04	-0.04	-0.04	-0.04	-0.04	-0.04	-0.04	-0.04	-0.05	-0.06
2016	Mid-Upper Keys Transition	1295	7	7	-0.10	-0.03	-0.13	-0.13	-0.12	-0.13	-0.13	-0.13	-0.14	-0.16	-0.16	-0.18	-0.20	-0.22
2018	Mid-Upper Keys Transition	1348	8	2	-0.19	-0.13	-0.14	-0.14	-0.14	-0.14	-0.14	-0.14	-0.15	-0.15	-0.15	-0.16	-0.19	-0.22
2016	Mid-Upper Keys Transition	1297	9	6	-0.37	-0.14	-0.25	-0.25	-0.24	-0.25	-0.25	-0.25	-0.26	-0.28	-0.28	-0.31	-0.33	-0.35
2016	Mid-Upper Keys Transition	1298	9	4	-0.13	-0.07	-0.11	-0.11	-0.10	-0.11	-0.11	-0.11	-0.11	-0.13	-0.12	-0.14	-0.16	-0.18
2016	Mid-Upper Keys Transition	1299	10	4	-1.57	-0.55	-0.62	-0.62	-0.61	-0.63	-0.62	-0.63	-0.63	-0.65	-0.65	-0.67	-0.68	-0.71
2016	Mid-Upper Keys Transition	1345	12	3	-0.12	-0.05	-0.10	-0.10	-0.09	-0.10	-0.10	-0.10	-0.10	-0.12	-0.12	-0.13	-0.15	-0.18
2016	Mid-Upper Keys Transition	1330	12	8	-2.26	-0.94	-1.05	-1.05	-1.04	-1.05	-1.04	-1.05	-1.06	-1.08	-1.08	-1.10	-1.13	-1.15
2018	Middle Keys	1286	3	1	-1.09	-0.40	-0.41	-0.41	-0.41	-0.41	-0.41	-0.41	-0.41	-0.42	-0.41	-0.42	-0.44	-0.46
2016	Middle Keys	1310	4	0	-0.20	-0.07	-0.07	-0.07	-0.07	-0.07	-0.07	-0.07	-0.07	-0.07	-0.07	-0.08	-0.09	-0.11
2018	Middle Keys	1154	4	1	-0.16	-0.07	-0.09	-0.09	-0.09	-0.10	-0.10	-0.10	-0.10	-0.11	-0.11	-0.12	-0.13	-0.16
2016	Middle Keys	1285	5	5	-0.92	-0.32	-0.42	-0.42	-0.41	-0.42	-0.42	-0.42	-0.43	-0.45	-0.45	-0.47	-0.50	-0.53
2016	Middle Keys	1259	5	5	-0.06	-0.03	-0.11	-0.11	-0.11	-0.11	-0.11	-0.11	-0.12	-0.14	-0.14	-0.16	-0.18	-0.21
2018	Middle Keys	1050	5	22	0.89	0.43	0.01	0.02	0.03	0.01	0.02	0.02	-0.02	-0.10	-0.11	-0.18	-0.20	-0.23
2018	Middle Keys	1053	6	22	0.65	0.35	-0.05	-0.05	-0.04	-0.06	-0.04	-0.05	-0.09	-0.16	-0.17	-0.23	-0.25	-0.28
2016	Middle Keys	1334	7	7	0.04	0.02	-0.08	-0.08	-0.07	-0.08	-0.08	-0.08	-0.09	-0.11	-0.11	-0.13	-0.15	-0.18
2018	Middle Keys	1552	7	18	0.80	0.37	0.03	0.04	0.05	0.03	0.04	0.04	0.00	-0.06	-0.06	-0.12	-0.14	-0.17
2016	Middle Keys	1315	7	8	-0.87	-0.29	-0.41	-0.40	-0.39	-0.40	-0.40	-0.40	-0.41	-0.44	-0.44	-0.46	-0.48	-0.50
2016	Middle Keys	1250	9	4	0.16	0.07	-0.03	-0.03	-0.02	-0.03	-0.02	-0.02	-0.03	-0.05	-0.05	-0.07	-0.08	-0.10
2018	Middle Keys	1236	10	11	0.40	0.18	-0.02	-0.01	0.00	-0.02	-0.01	-0.01	-0.03	-0.07	-0.07	-0.11	-0.13	-0.15
2016	Middle Keys	1303	11	9	0.11	0.06	-0.07	-0.07	-0.06	-0.07	-0.07	-0.08	-0.08	-0.11	-0.11	-0.14	-0.16	-0.18
2016	Middle Keys	1317	12	5	0.03	0.02	-0.07	-0.07	-0.06	-0.07	-0.06	-0.07	-0.07	-0.09	-0.09	-0.11	-0.13	-0.15
2018	Middle Keys	1345	12	5	-0.83	-0.35	-0.43	-0.43	-0.42	-0.43	-0.43	-0.43	-0.44	-0.46	-0.46	-0.48	-0.50	-0.53
2018	Lower Keys	1256	1	4	-2.71	-1.14	-1.23	-1.23	-1.22	-1.23	-1.23	-1.23	-1.24	-1.26	-1.26	-1.28	-1.30	-1.33
2016	Lower Keys	1395	2	0	-0.24	-0.08	-0.09	-0.09	-0.08	-0.09	-0.09	-0.09	-0.09	-0.09	-0.09	-0.10	-0.11	-0.13
2016	Lower Keys	1354	2	2	-0.18	-0.06	-0.18	-0.18	-0.17	-0.18	-0.18	-0.18	-0.19	-0.22	-0.22	-0.25	-0.27	-0.30
2018	Lower Keys	1004	3	0	-0.24	-0.08	-0.08	-0.09	-0.08	-0.08	-0.09	-0.09	-0.09	-0.09	-0.09	-0.10	-0.11	-0.13
2016	Lower Keys	1434	3	0	-0.24	-0.08	-0.09	-0.09	-0.09	-0.09	-0.09	-0.09	-0.09	-0.09	-0.09	-0.10	-0.11	-0.13
2018	Lower Keys	1257	4	12	-0.85	-0.22	-0.51	-0.51	-0.49	-0.51	-0.50	-0.51	-0.54	-0.59	-0.60	-0.65	-0.68	-0.70
2018	Lower Keys	1274	4	2	-0.63	-0.29	-0.32	-0.32	-0.31	-0.32	-0.32	-0.32	-0.32	-0.33	-0.33	-0.35	-0.37	-0.40
2016	Lower Keys	1409	5	1	-2.47	-0.89	-0.90	-0.90	-0.89	-0.90	-0.90	-0.90	-0.90	-0.90	-0.90	-0.91	-0.93	-0.96

2016	Lower Keys	1398	5	0	-0.06	-0.02	-0.02	-0.02	-0.02	-0.02	-0.02	-0.02	-0.02	-0.02	-0.02	-0.02	-0.03	-0.03
2016	Lower Keys	1401	5	5	-0.53	-0.17	-0.28	-0.29	-0.27	-0.28	-0.28	-0.28	-0.29	-0.32	-0.31	-0.34	-0.36	-0.38
2016	Lower Keys	1410	5	2	-1.17	-0.51	-0.55	-0.55	-0.54	-0.55	-0.55	-0.55	-0.55	-0.56	-0.56	-0.58	-0.60	-0.63
2016	Lower Keys	1416	6	4	-2.56	-1.08	-1.15	-1.15	-1.14	-1.15	-1.15	-1.15	-1.16	-1.18	-1.17	-1.19	-1.22	-1.25
2018	Lower Keys	1030	6	21	1.08	0.52	0.03	0.02	0.07	0.03	0.06	0.04	0.01	-0.09	-0.09	-0.17	-0.19	-0.21
2018	Lower Keys	1008	6	20	0.86	0.44	0.00	-0.02	0.01	-0.02	-0.01	-0.02	-0.06	-0.15	-0.15	-0.23	-0.25	-0.28
2018	Lower Keys	1047	6	7	0.12	0.06	-0.05	-0.06	-0.05	-0.06	-0.06	-0.06	-0.07	-0.10	-0.10	-0.12	-0.14	-0.17
2018	Lower Keys	1275	6	4	-0.46	-0.20	-0.27	-0.27	-0.27	-0.27	-0.27	-0.28	-0.28	-0.30	-0.30	-0.32	-0.35	-0.38
2018	Lower Keys	1020	6	32	1.10	0.54	0.04	0.01	0.05	0.01	0.03	0.01	-0.03	-0.13	-0.13	-0.22	-0.24	-0.27
2018	Lower Keys	1231	6	3	-0.11	-0.06	-0.11	-0.11	-0.11	-0.12	-0.12	-0.12	-0.12	-0.14	-0.13	-0.15	-0.18	-0.21
2016	Lower Keys	1422	7	17	-1.35	-0.42	-0.65	-0.65	-0.63	-0.65	-0.64	-0.64	-0.66	-0.71	-0.71	-0.75	-0.77	-0.80
2018	Lower Keys	1043	7	10	0.48	0.23	0.00	0.00	0.02	0.00	0.00	0.00	-0.02	-0.07	-0.07	-0.11	-0.13	-0.15
2016	Lower Keys	1377	7	9	0.25	0.12	-0.04	-0.05	-0.03	-0.05	-0.04	-0.04	-0.06	-0.09	-0.09	-0.12	-0.14	-0.16
2016	Lower Keys	1412	7	22	0.13	0.34	-0.55	-0.57	-0.50	-0.57	-0.52	-0.55	-0.62	-0.77	-0.79	-0.93	-0.97	-1.00
2016	Lower Keys	1373	8	31	1.25	0.60	0.07	0.05	0.09	0.05	0.08	0.07	0.02	-0.07	-0.08	-0.17	-0.19	-0.21
2016	Lower Keys	1427	8	0	-0.15	-0.05	-0.05	-0.05	-0.05	-0.05	-0.05	-0.06	-0.06	-0.06	-0.06	-0.06	-0.07	-0.08
2016	Lower Keys	1371	8	36	2.50	1.16	0.28	0.26	0.33	0.28	0.29	0.26	0.19	0.02	0.02	-0.13	-0.15	-0.18
2018	Lower Keys	1172	8	9	0.35	0.17	-0.01	-0.02	0.00	-0.02	-0.01	-0.02	-0.03	-0.06	-0.07	-0.10	-0.12	-0.14
2016	Lower Keys	1363	8	11	0.28	0.15	-0.05	-0.06	-0.04	-0.06	-0.04	-0.05	-0.07	-0.11	-0.11	-0.14	-0.16	-0.17
2016	Lower Keys	1431	8	12	0.46	0.22	-0.01	-0.01	0.01	-0.01	0.00	-0.01	-0.02	-0.07	-0.07	-0.11	-0.13	-0.15
2016	Lower Keys	1376	8	2	-0.18	-0.10	-0.11	-0.11	-0.11	-0.11	-0.11	-0.11	-0.11	-0.12	-0.12	-0.13	-0.15	-0.17
2016	Lower Keys	1379	9	17	0.48	0.25	-0.04	-0.06	-0.03	-0.06	-0.03	-0.04	-0.07	-0.12	-0.12	-0.17	-0.19	-0.21
2018	Lower Keys	1605	9	5	0.05	0.03	-0.05	-0.05	-0.04	-0.05	-0.05	-0.05	-0.06	-0.07	-0.07	-0.09	-0.10	-0.11
2018	Lower Keys	1106	10	15	0.65	0.30	0.01	0.01	0.03	0.01	0.02	0.01	-0.02	-0.07	-0.08	-0.13	-0.15	-0.17
2016	Lower Keys	1391	10	16	-0.38	-0.02	-0.43	-0.44	-0.40	-0.44	-0.41	-0.42	-0.46	-0.53	-0.54	-0.61	-0.63	-0.66
2018	Lower Keys	1031	11	0	0.04	0.02	0.01	0.01	0.01	0.01	0.01	0.01	0.01	0.01	0.01	0.01	0.00	-0.01
2016	Lower Keys	1404	11	10	0.13	0.09	-0.10	-0.11	-0.09	-0.11	-0.10	-0.10	-0.12	-0.16	-0.16	-0.19	-0.21	-0.23
2016	Lower Keys	1451	12	14	0.44	0.24	-0.09	-0.10	-0.07	-0.09	-0.07	-0.08	-0.11	-0.17	-0.18	-0.23	-0.25	-0.28
2016	Lower Keys	1438	12	4	-0.33	-0.12	-0.17	-0.17	-0.17	-0.17	-0.17	-0.17	-0.18	-0.19	-0.19	-0.21	-0.22	-0.25
Mexico																		
2021	Veracruz	Verde 4	3	32	-3.51	-0.21	-1.37	-1.37	-1.38	-1.41	-1.44	-1.45	-1.47	-1.50	-1.49	-1.52	-1.59	-1.67
2021	Veracruz	Sacrificios 7	4	15	0.31	0.26	-0.04	-0.05	-0.05	-0.09	-0.11	-0.13	-0.14	-0.17	-0.16	-0.19	-0.25	-0.32
2021	Veracruz	Blanca 4	4	38	-0.69	0.63	-0.89	-0.90	-0.90	-0.93	-0.96	-0.98	-0.99	-1.02	-1.01	-1.05	-1.11	-1.19

2021	Veracruz	Galleguilla 1	6	6	-1.56	-0.28	-0.33	-0.34	-0.34	-0.38	-0.41	-0.43	-0.44	-0.48	-0.47	-0.50	-0.57	-0.65
2021	Veracruz	Santiaguillo 5	7	36	-6.75	-0.78	-2.81	-2.82	-2.82	-2.86	-2.89	-2.91	-2.93	-2.96	-2.95	-2.99	-3.07	-3.15
2021	Veracruz	Isla de En medio 9	8	9	-0.54	-0.13	-0.20	-0.21	-0.21	-0.25	-0.27	-0.29	-0.30	-0.33	-0.32	-0.35	-0.41	-0.48
2021	Veracruz	Blanquilla 10	9	31	1.46	0.87	-0.20	-0.20	-0.21	-0.24	-0.27	-0.28	-0.30	-0.33	-0.32	-0.36	-0.42	-0.51
2021	Veracruz	Ahogado chico	9	26	0.53	0.63	-0.44	-0.44	-0.45	-0.48	-0.51	-0.52	-0.54	-0.57	-0.56	-0.59	-0.66	-0.74
2021	Veracruz	Verde 10	9	37	1.78	1.26	-0.40	-0.41	-0.42	-0.45	-0.48	-0.49	-0.51	-0.54	-0.53	-0.56	-0.63	-0.71
2021	Veracruz	Pajaros 11	10	18	-0.36	0.23	-0.32	-0.32	-0.33	-0.36	-0.39	-0.40	-0.42	-0.45	-0.44	-0.47	-0.54	-0.62
2021	Banco de Campeche	El vapor cresta	1	32	-0.84	0.69	-1.14	-1.15	-1.16	-1.19	-1.23	-1.24	-1.26	-1.29	-1.28	-1.32	-1.39	-1.46
2021	Banco de Campeche	Cayo Arenas Acropora	2	33	0.13	0.71	-1.02	-1.02	-1.03	-1.07	-1.10	-1.12	-1.14	-1.17	-1.16	-1.20	-1.29	-1.37
2021	Banco de Campeche	Tweed	6	24	-1.80	-0.41	-1.07	-1.07	-1.08	-1.11	-1.14	-1.15	-1.17	-1.20	-1.18	-1.22	-1.28	-1.34
2017	Banco de Campeche	Cayo_Arenas_5	8	21	-3.45	-0.82	-1.52	-1.53	-1.54	-1.57	-1.61	-1.62	-1.64	-1.67	-1.66	-1.70	-1.76	-1.83
2023	Banco de Campeche	Cayo_Arenas_15	8	23	-7.17	-1.45	-3.00	-3.00	-3.01	-3.04	-3.08	-3.09	-3.11	-3.14	-3.13	-3.17	-3.25	-3.33
2023	Banco de Campeche	Cayo Arenas 1	9	36	2.20	1.57	-0.81	-0.81	-0.82	-0.85	-0.88	-0.89	-0.91	-0.94	-0.93	-0.96	-1.03	-1.10
2021	Banco de Campeche	La pared del tata	10	14	0.13	0.14	-0.10	-0.10	-0.11	-0.14	-0.17	-0.18	-0.20	-0.22	-0.22	-0.25	-0.30	-0.36
2017	Banco de Campeche	Cayo_Arenas_6	10	27	1.32	1.20	-0.88	-0.89	-0.90	-0.93	-0.97	-0.98	-1.00	-1.03	-1.02	-1.06	-1.14	-1.22
2017	Banco de Campeche	Cayo_Arenas_11	10	27	-7.35	-1.55	-2.56	-2.57	-2.58	-2.61	-2.65	-2.66	-2.68	-2.71	-2.70	-2.74	-2.82	-2.90
2023	Banco de Campeche	Cayo_Arenas_16	10	16	-2.55	-0.55	-1.13	-1.14	-1.14	-1.17	-1.20	-1.22	-1.23	-1.26	-1.25	-1.28	-1.34	-1.40
2021	Banco de Campeche	Cayo_Arenas_13	12	63	6.97	3.95	-0.05	-0.05	-0.06	-0.10	-0.13	-0.15	-0.17	-0.20	-0.19	-0.23	-0.30	-0.37
2021	Banco de Campeche	Alacranes centro	12	16	0.44	0.37	-0.18	-0.18	-0.19	-0.22	-0.25	-0.27	-0.28	-0.32	-0.31	-0.35	-0.44	-0.54
2021	Banco de Campeche	Fondeadero	12	18	-0.20	0.14	-0.48	-0.48	-0.49	-0.52	-0.55	-0.56	-0.58	-0.61	-0.60	-0.64	-0.71	-0.79
2023	Banco de Campeche	Bajo Tortugas	12	30	2.16	1.49	-0.51	-0.52	-0.53	-0.56	-0.59	-0.60	-0.62	-0.65	-0.63	-0.67	-0.74	-0.82
2018	Northern Quintana Roo	Radio Pirata	1	21	0.47	0.34	-0.20	-0.20	-0.21	-0.24	-0.27	-0.29	-0.31	-0.34	-0.32	-0.36	-0.42	-0.48
2017	Northern Quintana Roo	Bonanza	1	9	0.31	0.16	0.05	0.04	0.03	0.00	-0.03	-0.04	-0.06	-0.08	-0.07	-0.11	-0.16	-0.22
2017	Northern Quintana Roo	Ixlaché	1	16	0.20	0.30	-0.38	-0.39	-0.40	-0.43	-0.47	-0.48	-0.50	-0.53	-0.52	-0.56	-0.62	-0.69
2019	Northern Quintana Roo	Mar P1	1	13	-1.44	-0.34	-0.27	-0.28	-0.28	-0.32	-0.35	-0.36	-0.38	-0.41	-0.40	-0.44	-0.50	-0.57
2018	Northern Quintana Roo	Nizuc C3	2	43	3.03	2.66	-0.90	-0.91	-0.91	-0.95	-0.98	-1.00	-1.02	-1.05	-1.04	-1.08	-1.14	-1.22

2017	Northern Quintana Roo	Limones	3	60	13.80	8.01	-0.83	-0.84	-0.85	-0.89	-0.92	-0.94	-0.96	-0.99	-0.98	-1.02	-1.10	-1.18
2018	Northern Quintana Roo	Mar F2	5	25	-0.57	0.15	-0.38	-0.38	-0.39	-0.42	-0.46	-0.47	-0.49	-0.52	-0.51	-0.55	-0.62	-0.70
2019	Northern Quintana Roo	Nizuc 5m	5	8	-0.87	-0.21	-0.16	-0.17	-0.17	-0.21	-0.24	-0.25	-0.27	-0.30	-0.29	-0.33	-0.40	-0.47
2019	Northern Quintana Roo	La Catedral 5m	5	10	-1.23	-0.38	-0.41	-0.41	-0.42	-0.45	-0.49	-0.50	-0.52	-0.55	-0.54	-0.58	-0.66	-0.74
2019	Northern Quintana Roo	Manchones	6	13	-5.66	-1.43	-1.91	-1.92	-1.92	-1.96	-1.99	-2.01	-2.03	-2.06	-2.05	-2.09	-2.16	-2.25
2019	Northern Quintana Roo	Mar F5	6	24	-2.02	-0.44	-1.19	-1.20	-1.20	-1.24	-1.27	-1.29	-1.31	-1.34	-1.33	-1.38	-1.47	-1.58
2019	Northern Quintana Roo	Cuevones	7	21	-3.72	-0.77	-1.56	-1.57	-1.57	-1.61	-1.65	-1.66	-1.68	-1.72	-1.70	-1.75	-1.82	-1.91
2018	Northern Quintana Roo	Dicks	7	13	-4.36	-1.00	-1.14	-1.15	-1.16	-1.19	-1.22	-1.24	-1.26	-1.28	-1.27	-1.31	-1.37	-1.44
2018	Northern Quintana Roo	Tanchacte	7	30	-0.09	0.24	-0.64	-0.65	-0.66	-0.69	-0.72	-0.74	-0.76	-0.79	-0.78	-0.82	-0.89	-0.96
2018	Northern Quintana Roo	Manchones Norte	10	35	1.85	1.08	0.00	0.00	-0.01	-0.05	-0.08	-0.10	-0.12	-0.15	-0.14	-0.18	-0.25	-0.33
2019	Northern Quintana Roo	Bonanza Profundo	10	11	-0.33	-0.04	-0.24	-0.25	-0.26	-0.29	-0.32	-0.34	-0.35	-0.38	-0.37	-0.41	-0.46	-0.53
2018	Northern Quintana Roo	Bocana Caricomp	10	5	-0.27	-0.09	0.07	0.07	0.06	0.03	0.00	-0.01	-0.03	-0.06	-0.05	-0.08	-0.14	-0.21
2018	Northern Quintana Roo	PM-F2	10	12	0.10	0.12	0.05	0.04	0.04	0.00	-0.03	-0.04	-0.06	-0.09	-0.08	-0.11	-0.17	-0.24
2019	Northern Quintana Roo	La Catedral Posterior	10	17	-0.20	0.40	-0.72	-0.73	-0.73	-0.77	-0.80	-0.82	-0.84	-0.87	-0.86	-0.90	-0.96	-1.03
2019	Northern Quintana Roo	Punta Maroma Norte	10	17	-0.44	0.00	-0.47	-0.47	-0.48	-0.51	-0.55	-0.56	-0.58	-0.61	-0.60	-0.64	-0.71	-0.79
2021	Northern Quintana Roo	Akumal Langosta	10	10	-3.70	-0.94	-1.01	-1.01	-1.02	-1.05	-1.09	-1.10	-1.12	-1.15	-1.14	-1.18	-1.24	-1.31
2018	Northern Quintana Roo	Mar F4	10	16	0.38	0.23	0.06	0.05	0.05	0.01	-0.02	-0.04	-0.06	-0.09	-0.07	-0.11	-0.17	-0.24
2018	Northern Quintana Roo	Nizuc F3	11	7	0.08	0.06	0.09	0.08	0.08	0.04	0.01	-0.01	-0.03	-0.06	-0.04	-0.08	-0.14	-0.21
2018	Northern Quintana Roo	Yalku	11	23	0.69	0.60	-0.28	-0.28	-0.29	-0.33	-0.36	-0.38	-0.40	-0.43	-0.42	-0.46	-0.54	-0.63
2019	Northern Quintana Roo	Punta Maroma Sur	11	29	-0.58	-0.07	-0.54	-0.54	-0.55	-0.58	-0.62	-0.63	-0.65	-0.68	-0.67	-0.70	-0.77	-0.84
2018	Northern Quintana Roo	Media Luna	11	19	1.15	0.76	-0.18	-0.19	-0.19	-0.23	-0.27	-0.28	-0.30	-0.33	-0.32	-0.36	-0.44	-0.53
2018	Cozumel	Punta Sur Somero B	3	25	4.21	2.42	-0.32	-0.33	-0.33	-0.37	-0.41	-0.42	-0.45	-0.48	-0.47	-0.52	-0.62	-0.73
2019	Cozumel	Microatolones 5m	5	6	-0.73	-0.20	-0.14	-0.14	-0.15	-0.18	-0.22	-0.23	-0.25	-0.28	-0.27	-0.31	-0.37	-0.45
2017	Cozumel	Palancar Jardines	6	34	1.06	0.86	-0.73	-0.73	-0.74	-0.77	-0.80	-0.81	-0.83	-0.86	-0.86	-0.89	-0.94	-1.02
2018	Cozumel	Colombia Somero	6	43	1.21	0.89	-0.34	-0.34	-0.35	-0.39	-0.42	-0.44	-0.46	-0.49	-0.48	-0.53	-0.62	-0.72

2017	Cozumel	Paraiso	8	11	0.15	0.15	-0.02	-0.02	-0.03	-0.06	-0.10	-0.11	-0.13	-0.16	-0.15	-0.19	-0.27	-0.37
2018	Cozumel	Hanan	11	17	-0.20	0.00	-0.16	-0.17	-0.17	-0.21	-0.24	-0.26	-0.28	-0.31	-0.30	-0.34	-0.40	-0.48
2018	Cozumel	Caracolillo	11	23	-0.81	0.01	-0.67	-0.67	-0.68	-0.72	-0.76	-0.78	-0.80	-0.85	-0.83	-0.90	-1.06	-1.24
2017	Cozumel	Chankanaab	12	19	0.29	0.35	-0.38	-0.39	-0.40	-0.43	-0.47	-0.48	-0.50	-0.54	-0.53	-0.57	-0.65	-0.74
2018	Cozumel	MX3054	12	15	-1.71	-0.55	-0.75	-0.76	-0.77	-0.80	-0.83	-0.85	-0.86	-0.89	-0.88	-0.92	-0.98	-1.05
2018	Central Quintana Roo (Sian Ka'an)	San Antonio Posterior	1	9	-0.08	0.08	-0.04	-0.04	-0.05	-0.09	-0.12	-0.14	-0.16	-0.19	-0.18	-0.22	-0.29	-0.37
2018	Central Quintana Roo (Sian Ka'an)	Yuyum Posterior	1	10	-1.34	-0.43	-0.49	-0.49	-0.50	-0.53	-0.56	-0.57	-0.59	-0.62	-0.61	-0.64	-0.70	-0.76
2018	Central Quintana Roo (Sian Ka'an)	Niccehabin Posterior	2	36	0.96	0.72	-0.38	-0.39	-0.39	-0.43	-0.47	-0.48	-0.50	-0.54	-0.53	-0.57	-0.66	-0.76
2018	Central Quintana Roo (Sian Ka'an)	Niccehabin 6m	6	16	0.33	0.25	-0.10	-0.10	-0.11	-0.14	-0.18	-0.19	-0.21	-0.24	-0.23	-0.26	-0.33	-0.41
2018	Central Quintana Roo (Sian Ka'an)	Yuyum 6m	6	3	-0.23	-0.11	0.11	0.10	0.10	0.06	0.03	0.01	-0.01	-0.04	-0.03	-0.07	-0.13	-0.19
2018	Central Quintana Roo (Sian Ka'an)	San Antonio 6m	6	20	0.13	0.18	-0.13	-0.14	-0.14	-0.18	-0.21	-0.22	-0.24	-0.27	-0.26	-0.30	-0.36	-0.44
2018	Central Quintana Roo (Sian Ka'an)	Punta Allen Norte	10	18	0.49	0.28	0.03	0.02	0.02	-0.02	-0.05	-0.06	-0.08	-0.11	-0.10	-0.13	-0.20	-0.27
2018	Central Quintana Roo (Sian Ka'an)	Yuyum Frontal Somero	10	10	0.18	0.11	0.08	0.08	0.07	0.03	0.00	-0.02	-0.03	-0.06	-0.05	-0.09	-0.15	-0.22
2018	Central Quintana Roo (Sian Ka'an)	Punta Allen Centro	11	22	1.42	0.76	0.00	-0.01	-0.02	-0.05	-0.08	-0.10	-0.12	-0.15	-0.13	-0.17	-0.24	-0.32
2018	Central Quintana Roo (Sian Ka'an)	Niccehabin Frontal Somero	12	14	0.62	0.33	0.07	0.06	0.05	0.02	-0.01	-0.03	-0.04	-0.07	-0.06	-0.10	-0.15	-0.22
2019	Banco Chinchorro	CHI05	5	25	-0.55	0.23	-0.56	-0.57	-0.58	-0.62	-0.65	-0.67	-0.69	-0.72	-0.71	-0.75	-0.83	-0.92
2019	Banco Chinchorro	MX3082	6	38	5.26	2.84	-0.69	-0.69	-0.70	-0.73	-0.76	-0.77	-0.79	-0.82	-0.81	-0.85	-0.93	-1.02
2019	Banco Chinchorro	CHI04	12	9	-0.53	-0.14	-0.22	-0.23	-0.23	-0.27	-0.30	-0.31	-0.33	-0.36	-0.35	-0.39	-0.45	-0.53
2019	Banco Chinchorro	Far Star	12	16	0.01	0.09	-0.04	-0.04	-0.05	-0.08	-0.11	-0.13	-0.15	-0.17	-0.16	-0.20	-0.26	-0.33
2019	Banco Chinchorro	Isla Che	12	15	0.27	0.25	-0.11	-0.12	-0.12	-0.16	-0.20	-0.21	-0.23	-0.26	-0.25	-0.29	-0.36	-0.44
2019	Banco Chinchorro	La Baliza 2	12	10	-1.04	-0.28	-0.41	-0.42	-0.42	-0.46	-0.49	-0.51	-0.53	-0.56	-0.55	-0.60	-0.70	-0.83

2019	Southern Quintana Roo	Mahahual Cresta	1	8	-11.07	-2.51	-2.51	-2.51	-2.52	-2.55	-2.58	-2.59	-2.61	-2.64	-2.62	-2.66	-2.71	-2.77
2019	Southern Quintana Roo	Portillas Cresta	1	19	-0.46	-0.03	-0.13	-0.14	-0.14	-0.18	-0.21	-0.22	-0.24	-0.27	-0.26	-0.31	-0.40	-0.50
2019	Southern Quintana Roo	Quebrado 5m	5	14	-1.79	-0.28	-0.54	-0.54	-0.55	-0.58	-0.62	-0.63	-0.65	-0.68	-0.67	-0.72	-0.80	-0.91
2019	Southern Quintana Roo	Portillas 5m	5	17	-1.34	-0.24	-0.41	-0.41	-0.42	-0.45	-0.49	-0.50	-0.52	-0.55	-0.54	-0.58	-0.65	-0.73
2017	Southern Quintana Roo	Mahahual Centro	6	8	-0.30	-0.06	-0.11	-0.12	-0.12	-0.16	-0.19	-0.20	-0.22	-0.25	-0.24	-0.28	-0.33	-0.41
2017	Southern Quintana Roo	40 Cañones	6	18	1.58	0.92	-0.05	-0.06	-0.06	-0.10	-0.13	-0.15	-0.17	-0.20	-0.19	-0.23	-0.30	-0.38
2017	Southern Quintana Roo	El Faro	7	10	-0.40	-0.07	-0.22	-0.22	-0.23	-0.26	-0.30	-0.31	-0.33	-0.36	-0.34	-0.38	-0.44	-0.50
2017	Southern Quintana Roo	Hotel Arenas	7	10	-0.27	-0.03	-0.07	-0.08	-0.08	-0.12	-0.15	-0.16	-0.18	-0.21	-0.20	-0.24	-0.30	-0.37
2017	Southern Quintana Roo	Puerto Angel	9	10	0.09	0.09	-0.01	-0.01	-0.02	-0.05	-0.08	-0.10	-0.12	-0.14	-0.13	-0.17	-0.22	-0.29
2017	Southern Quintana Roo	Faro Viejo	10	12	0.12	0.14	-0.10	-0.11	-0.11	-0.15	-0.18	-0.19	-0.21	-0.24	-0.23	-0.27	-0.33	-0.40
2019	Southern Quintana Roo	Bacalar Chico	12	18	-0.02	0.17	-0.16	-0.16	-0.17	-0.20	-0.24	-0.25	-0.27	-0.30	-0.29	-0.33	-0.40	-0.49
2019	Southern Quintana Roo	Portillas	12	12	-0.72	-0.12	-0.37	-0.38	-0.38	-0.42	-0.45	-0.46	-0.48	-0.51	-0.50	-0.54	-0.61	-0.69
2017	Southern Quintana Roo	Mahahual	12	11	0.00	0.17	-0.36	-0.37	-0.37	-0.40	-0.44	-0.45	-0.47	-0.50	-0.49	-0.53	-0.59	-0.67
Bonaire																		
2017	North_Exposed	71	6	8.3	-0.54	-0.16	-0.31	-0.32	-0.28	-0.32	-0.32	-0.32	-0.32	-0.34	-0.37	-0.40	-0.42	-0.46
2017	North_Exposed	72	6	1.98	-0.95	-0.34	-0.38	-0.38	-0.37	-0.38	-0.38	-0.38	-0.38	-0.39	-0.40	-0.42	-0.45	-0.50
2017	North_Exposed	73	6	23.39	-0.44	0.04	-0.46	-0.50	-0.37	-0.49	-0.47	-0.47	-0.48	-0.53	-0.63	-0.71	-0.75	-0.77
2017	North_Exposed	74	6	6.48	-0.69	-0.26	-0.37	-0.37	-0.35	-0.37	-0.37	-0.37	-0.37	-0.38	-0.40	-0.42	-0.44	-0.47
2017	North_Exposed	75	6	14.53	-0.39	0.02	-0.33	-0.36	-0.26	-0.35	-0.34	-0.34	-0.34	-0.39	-0.46	-0.52	-0.57	-0.63
2017	North_Exposed	76	6	16.02	0.40	0.41	-0.26	-0.30	-0.13	-0.29	-0.27	-0.27	-0.27	-0.35	-0.48	-0.58	-0.63	-0.67
2017	North_Exposed	77	6	2.52	-1.18	-0.42	-0.50	-0.51	-0.48	-0.51	-0.51	-0.51	-0.50	-0.52	-0.54	-0.56	-0.59	-0.63
2017	North_Exposed	78	6	0.55	-1.15	-0.42	-0.43	-0.43	-0.42	-0.43	-0.43	-0.43	-0.43	-0.44	-0.44	-0.45	-0.47	-0.50
2017	North_Exposed	79	6	10.33	-1.09	-0.34	-0.54	-0.56	-0.50	-0.55	-0.55	-0.55	-0.55	-0.58	-0.62	-0.65	-0.68	-0.72
2017	North_Exposed	80	6	33.49	1.42	0.82	-0.01	-0.08	0.15	-0.06	-0.02	-0.03	-0.04	-0.13	-0.30	-0.41	-0.46	-0.48
2017	North_Exposed	81	6	6.66	-0.18	-0.03	-0.18	-0.19	-0.15	-0.19	-0.19	-0.19	-0.19	-0.20	-0.24	-0.26	-0.28	-0.31
2017	North_Exposed	82	6	22.9	-0.27	0.08	-0.46	-0.50	-0.36	-0.49	-0.47	-0.47	-0.47	-0.53	-0.65	-0.72	-0.76	-0.79
2017	North_Exposed	83	6	9.69	0.99	0.56	0.03	-0.01	0.13	0.00	0.02	0.02	0.02	-0.04	-0.15	-0.22	-0.26	-0.28
2017	North_Exposed	84	6	3.61	-1.37	-0.57	-0.64	-0.65	-0.63	-0.65	-0.65	-0.65	-0.65	-0.66	-0.68	-0.70	-0.72	-0.76
2017	North_Exposed	85	6	7.92	-1.31	-0.45	-0.74	-0.76	-0.68	-0.75	-0.74	-0.74	-0.74	-0.78	-0.83	-0.88	-0.91	-0.95

2017	North_Exposed	86	6	44.43	3.12	1.90	0.12	-0.01	0.46	0.03	0.10	0.08	0.07	-0.12	-0.47	-0.71	-0.78	-0.81
2017	North_Exposed	87	6	17.4	0.38	0.34	-0.22	-0.26	-0.11	-0.25	-0.22	-0.23	-0.23	-0.29	-0.41	-0.48	-0.52	-0.54
2017	North_Exposed	88	6	35.19	3.09	1.80	0.25	0.13	0.54	0.16	0.23	0.22	0.20	0.07	-0.27	-0.48	-0.54	-0.56
2017	North_Exposed	89	6	6.7	-1.59	-0.62	-0.74	-0.75	-0.72	-0.75	-0.74	-0.74	-0.74	-0.76	-0.78	-0.81	-0.83	-0.85
2017	North_Exposed	90	6	5.22	-0.22	-0.07	-0.17	-0.18	-0.15	-0.17	-0.17	-0.17	-0.17	-0.18	-0.21	-0.22	-0.23	-0.25
2017	North_Exposed	91	6	6.49	-0.89	-0.29	-0.52	-0.54	-0.48	-0.53	-0.52	-0.53	-0.53	-0.55	-0.60	-0.63	-0.66	-0.68
2017	North_Exposed	92	6	12.79	0.00	0.11	-0.24	-0.26	-0.17	-0.26	-0.24	-0.25	-0.25	-0.28	-0.36	-0.41	-0.43	-0.45
2017	North_Exposed	71	11	8.63	-0.01	0.05	-0.18	-0.19	-0.13	-0.19	-0.18	-0.18	-0.18	-0.21	-0.25	-0.29	-0.32	-0.34
2017	North_Exposed	72	11	12.71	0.25	0.18	-0.14	-0.16	-0.07	-0.15	-0.14	-0.14	-0.15	-0.18	-0.25	-0.29	-0.32	-0.34
2017	North_Exposed	73	11	12.34	0.02	0.09	-0.20	-0.22	-0.14	-0.21	-0.20	-0.20	-0.21	-0.24	-0.30	-0.34	-0.37	-0.40
2017	North_Exposed	74	11	9.18	-0.49	-0.14	-0.38	-0.40	-0.33	-0.39	-0.38	-0.38	-0.38	-0.41	-0.46	-0.50	-0.53	-0.56
2017	North_Exposed	75	11	10.75	-0.73	-0.19	-0.47	-0.49	-0.41	-0.48	-0.48	-0.48	-0.48	-0.51	-0.57	-0.62	-0.66	-0.70
2017	North_Exposed	76	11	9.23	-1.62	-0.63	-0.82	-0.83	-0.78	-0.83	-0.82	-0.83	-0.82	-0.85	-0.89	-0.92	-0.95	-0.99
2017	North_Exposed	77	11	7.47	-1.51	-0.55	-0.73	-0.75	-0.70	-0.74	-0.74	-0.74	-0.74	-0.76	-0.80	-0.83	-0.85	-0.87
2017	North_Exposed	78	11	6.51	-0.71	-0.26	-0.42	-0.43	-0.39	-0.43	-0.43	-0.43	-0.43	-0.45	-0.48	-0.50	-0.52	-0.55
2017	North_Exposed	79	11	8.45	-1.73	-0.64	-0.84	-0.86	-0.80	-0.85	-0.85	-0.85	-0.85	-0.87	-0.91	-0.95	-0.97	-0.99
2017	North_Exposed	80	11	4.84	-0.55	-0.21	-0.28	-0.28	-0.26	-0.28	-0.28	-0.28	-0.28	-0.29	-0.30	-0.31	-0.31	-0.32
2017	North_Exposed	81	11	21.16	0.05	0.19	-0.31	-0.35	-0.22	-0.34	-0.32	-0.32	-0.32	-0.38	-0.48	-0.55	-0.58	-0.60
2017	North_Exposed	82	11	27.7	1.24	1.09	-0.59	-0.71	-0.27	-0.68	-0.61	-0.62	-0.63	-0.81	-1.15	-1.37	-1.45	-1.48
2017	North_Exposed	83	11	18.11	-0.76	-0.15	-0.64	-0.68	-0.55	-0.67	-0.65	-0.65	-0.65	-0.71	-0.81	-0.87	-0.91	-0.93
2017	North_Exposed	84	11	18.53	0.72	0.69	-0.49	-0.58	-0.27	-0.56	-0.51	-0.52	-0.53	-0.65	-0.89	-1.06	-1.13	-1.17
2017	North_Exposed	85	11	28.87	3.11	2.06	-0.02	-0.18	0.37	-0.13	-0.04	-0.06	-0.08	-0.30	-0.72	-0.99	-1.08	-1.11
2017	North_Exposed	86	11	24.5	3.23	1.86	0.27	0.15	0.57	0.18	0.25	0.23	0.22	0.05	-0.27	-0.48	-0.55	-0.59
2017	North_Exposed	87	11	40.58	6.55	3.61	0.95	0.75	1.45	0.81	0.92	0.89	0.87	0.60	0.06	-0.29	-0.40	-0.44
2017	North_Exposed	88	11	46.21	9.57	5.18	1.52	1.25	2.21	1.33	1.48	1.45	1.42	1.10	0.31	-0.17	-0.31	-0.34
2017	North_Exposed	89	11	50.51	8.25	4.59	1.10	0.85	1.76	0.92	1.07	1.04	1.01	0.71	-0.05	-0.51	-0.64	-0.67
2017	North_Exposed	90	11	48.68	7.98	4.43	1.10	0.85	1.73	0.92	1.06	1.04	1.01	0.72	-0.01	-0.44	-0.57	-0.60
2017	North_Exposed	91	11	36.72	7.33	4.16	0.83	0.59	1.46	0.66	0.80	0.77	0.74	0.45	-0.27	-0.71	-0.85	-0.89
2017	North_Exposed	92	11	19.26	0.72	0.69	-0.45	-0.54	-0.23	-0.51	-0.47	-0.48	-0.48	-0.59	-0.84	-1.00	-1.07	-1.12
2017	North	93	6	21.2	-0.17	0.18	-0.55	-0.61	-0.41	-0.59	-0.56	-0.57	-0.57	-0.64	-0.80	-0.90	-0.94	-0.97
2017	North	94	6	34.01	5.13	2.92	0.52	0.34	0.97	0.39	0.49	0.47	0.45	0.24	-0.28	-0.60	-0.70	-0.73
2017	North	95	6	25.07	0.74	0.63	-0.32	-0.39	-0.14	-0.37	-0.33	-0.34	-0.34	-0.43	-0.63	-0.76	-0.80	-0.83
2017	North	96	6	8.3	-1.16	-0.40	-0.63	-0.64	-0.58	-0.64	-0.63	-0.63	-0.63	-0.66	-0.71	-0.74	-0.76	-0.79

2017	North	97	6	36.73	4.41	2.64	0.44	0.27	0.85	0.32	0.41	0.40	0.38	0.19	-0.30	-0.59	-0.68	-0.71
2017	North	98	6	23.19	3.07	1.73	0.28	0.17	0.55	0.20	0.26	0.25	0.24	0.11	-0.21	-0.40	-0.46	-0.49
2017	North	99	6	26.44	3.42	1.63	0.44	0.35	0.67	0.38	0.43	0.42	0.41	0.30	0.04	-0.12	-0.17	-0.20
2017	North	100	6	22.11	2.30	1.29	0.30	0.23	0.49	0.25	0.29	0.28	0.27	0.18	-0.03	-0.17	-0.21	-0.23
2017	North	101	6	12.75	1.56	0.90	0.10	0.04	0.25	0.06	0.09	0.08	0.08	-0.01	-0.17	-0.28	-0.32	-0.35
2017	North	102	6	18.5	2.14	1.10	0.12	0.05	0.31	0.07	0.11	0.10	0.10	-0.01	-0.21	-0.34	-0.39	-0.41
2017	North	103	6	45.06	7.63	4.35	1.23	1.00	1.82	1.07	1.20	1.18	1.15	0.83	0.20	-0.20	-0.32	-0.34
2017	North	104	6	13.38	0.94	0.64	-0.13	-0.18	0.02	-0.17	-0.14	-0.14	-0.15	-0.23	-0.39	-0.49	-0.53	-0.56
2017	North	105	6	62.88	9.39	5.50	1.28	0.97	2.08	1.06	1.24	1.20	1.17	0.73	-0.11	-0.66	-0.82	-0.84
2017	North	93	11	33.05	5.27	2.64	0.49	0.33	0.90	0.38	0.47	0.45	0.43	0.24	-0.23	-0.52	-0.62	-0.67
2017	North	94	11	41.74	6.59	3.81	0.57	0.32	1.18	0.39	0.53	0.50	0.48	0.20	-0.51	-0.94	-1.07	-1.10
2017	North	95	11	37.22	7.05	3.99	0.89	0.66	1.47	0.72	0.85	0.83	0.80	0.53	-0.15	-0.56	-0.70	-0.76
2017	North	96	11	39.52	7.14	4.22	0.90	0.66	1.53	0.73	0.87	0.84	0.81	0.52	-0.20	-0.64	-0.78	-0.81
2017	North	97	11	47.92	11.07	5.99	1.64	1.31	2.46	1.41	1.59	1.55	1.52	1.14	0.19	-0.39	-0.57	-0.62
2017	North	98	11	28.79	3.07	1.98	0.15	0.01	0.50	0.05	0.13	0.11	0.10	-0.06	-0.47	-0.71	-0.80	-0.84
2017	North	99	11	0.93	-1.71	-0.57	-0.61	-0.61	-0.60	-0.61	-0.61	-0.61	-0.61	-0.62	-0.63	-0.65	-0.68	-0.74
2017	North	100	11	26.96	2.80	1.46	0.17	0.07	0.41	0.10	0.15	0.14	0.13	0.02	-0.26	-0.44	-0.50	-0.54
2017	North	101	11	27.02	3.62	2.14	0.21	0.07	0.57	0.11	0.19	0.17	0.16	-0.05	-0.43	-0.69	-0.78	-0.82
2017	North	102	11	27.56	3.29	2.05	0.16	0.02	0.52	0.06	0.14	0.13	0.11	-0.09	-0.47	-0.72	-0.81	-0.85
2017	North	103	11	38.85	6.50	3.67	0.77	0.55	1.32	0.62	0.74	0.71	0.69	0.39	-0.20	-0.58	-0.70	-0.73
2017	North	104	11	33.54	4.59	2.62	0.45	0.29	0.86	0.34	0.43	0.41	0.39	0.16	-0.27	-0.56	-0.66	-0.70
2017	North	105	11	27.55	2.33	1.72	-0.47	-0.63	-0.05	-0.58	-0.49	-0.51	-0.53	-0.76	-1.20	-1.49	-1.59	-1.63
2017	Klein Bonaire	55	6	14.3	-0.03	0.04	-0.12	-0.13	-0.09	-0.13	-0.12	-0.12	-0.12	-0.14	-0.17	-0.20	-0.21	-0.23
2017	Klein Bonaire	56	6	13.53	-0.75	-0.25	-0.44	-0.46	-0.40	-0.45	-0.45	-0.45	-0.45	-0.47	-0.51	-0.54	-0.56	-0.59
2017	Klein Bonaire	57	6	25.81	-0.75	0.14	-0.93	-1.01	-0.72	-0.98	-0.95	-0.95	-0.96	-1.08	-1.29	-1.45	-1.53	-1.59
2017	Klein Bonaire	58	6	41.1	-0.18	0.68	-1.30	-1.45	-0.93	-1.41	-1.33	-1.34	-1.36	-1.57	-1.97	-2.23	-2.32	-2.36
2017	Klein Bonaire	59	6	28.13	0.87	0.85	-0.47	-0.57	-0.22	-0.54	-0.49	-0.50	-0.51	-0.65	-0.92	-1.10	-1.16	-1.19
2017	Klein Bonaire	60	6	14.23	-0.01	0.15	-0.31	-0.34	-0.22	-0.33	-0.32	-0.32	-0.32	-0.37	-0.47	-0.54	-0.57	-0.60
2017	Klein Bonaire	61	6	6.19	-0.72	-0.27	-0.39	-0.40	-0.37	-0.40	-0.39	-0.39	-0.39	-0.41	-0.43	-0.45	-0.47	-0.49
2017	Klein Bonaire	62	6	16.57	-0.31	0.00	-0.39	-0.42	-0.32	-0.41	-0.40	-0.40	-0.40	-0.45	-0.52	-0.58	-0.61	-0.64
2017	Klein Bonaire	63	6	11.23	-0.04	0.06	-0.18	-0.20	-0.13	-0.19	-0.18	-0.19	-0.19	-0.21	-0.26	-0.30	-0.32	-0.35
2017	Klein Bonaire	64	6	9.55	-0.63	-0.16	-0.44	-0.46	-0.39	-0.46	-0.45	-0.45	-0.45	-0.48	-0.54	-0.58	-0.61	-0.63
2017	Klein Bonaire	65	6	18.71	-1.10	-0.28	-0.76	-0.80	-0.67	-0.79	-0.77	-0.77	-0.78	-0.83	-0.93	-0.99	-1.02	-1.04

2017	Klein Bonaire	118	6	2.79	-1.19	-0.43	-0.52	-0.53	-0.50	-0.52	-0.53	-0.52	-0.52	-0.54	-0.56	-0.58	-0.62	-0.67
2017	Klein Bonaire	119	6	18.82	1.54	0.96	-0.01	-0.08	0.17	-0.06	-0.02	-0.03	-0.04	-0.14	-0.34	-0.47	-0.52	-0.54
2017	Klein Bonaire	120	6	23.49	2.47	1.45	0.22	0.13	0.46	0.16	0.21	0.20	0.19	0.06	-0.19	-0.35	-0.40	-0.42
2017	Klein Bonaire	121	6	11.53	0.49	0.32	-0.02	-0.04	0.05	-0.03	-0.02	-0.02	-0.02	-0.06	-0.13	-0.17	-0.19	-0.20
2017	Klein Bonaire	122	6	7.99	5.00	3.89	1.25	1.05	1.75	1.11	1.22	1.20	1.18	0.91	0.37	0.03	-0.06	-0.07
2017	Klein Bonaire	123	6	0.93	-0.12	-0.04	-0.07	-0.07	-0.06	-0.07	-0.07	-0.07	-0.07	-0.07	-0.08	-0.08	-0.08	-0.09
2017	Klein Bonaire	124	6	11.83	0.03	0.21	-0.33	-0.37	-0.22	-0.35	-0.33	-0.34	-0.34	-0.40	-0.51	-0.58	-0.61	-0.62
2017	Klein Bonaire	125	6	14.19	8.77	6.89	2.13	1.78	3.03	1.88	2.09	2.04	2.00	1.52	0.56	-0.06	-0.22	-0.23
2017	Klein Bonaire	126	6	15.29	0.55	0.45	-0.14	-0.19	-0.03	-0.18	-0.15	-0.16	-0.16	-0.23	-0.35	-0.43	-0.48	-0.51
2017	Klein Bonaire	127	6	9.89	-1.43	-0.56	-0.71	-0.72	-0.68	-0.72	-0.72	-0.72	-0.72	-0.74	-0.77	-0.79	-0.82	-0.85
2017	Klein Bonaire	55	11	16.13	0.20	0.41	-0.44	-0.51	-0.28	-0.49	-0.46	-0.46	-0.47	-0.56	-0.73	-0.85	-0.91	-0.96
2017	Klein Bonaire	56	11	19.86	0.52	0.57	-0.43	-0.51	-0.24	-0.49	-0.45	-0.45	-0.46	-0.57	-0.77	-0.91	-0.96	-0.98
2017	Klein Bonaire	57	11	44.4	7.16	4.07	0.84	0.60	1.45	0.67	0.80	0.77	0.75	0.41	-0.24	-0.67	-0.80	-0.84
2017	Klein Bonaire	58	11	30.91	3.39	2.09	0.15	0.01	0.52	0.05	0.13	0.11	0.10	-0.11	-0.50	-0.76	-0.86	-0.91
2017	Klein Bonaire	59	11	39.48	4.91	3.06	0.34	0.14	0.86	0.20	0.32	0.29	0.27	-0.01	-0.56	-0.92	-1.03	-1.06
2017	Klein Bonaire	60	11	11.89	-0.04	0.06	-0.21	-0.23	-0.15	-0.22	-0.21	-0.21	-0.21	-0.24	-0.30	-0.34	-0.36	-0.39
2017	Klein Bonaire	61	11	14.09	0.20	0.21	-0.20	-0.24	-0.12	-0.23	-0.21	-0.21	-0.22	-0.26	-0.35	-0.41	-0.44	-0.46
2017	Klein Bonaire	62	11	25.17	1.78	1.11	0.02	-0.06	0.23	-0.03	0.01	0.00	-0.01	-0.12	-0.34	-0.49	-0.55	-0.59
2017	Klein Bonaire	63	11	10.22	-0.35	-0.04	-0.37	-0.40	-0.31	-0.39	-0.38	-0.38	-0.38	-0.42	-0.49	-0.54	-0.57	-0.60
2017	Klein Bonaire	64	11	17.44	0.77	0.50	-0.07	-0.11	0.04	-0.10	-0.07	-0.08	-0.08	-0.14	-0.26	-0.34	-0.37	-0.39
2017	Klein Bonaire	65	11	38.28	6.66	3.61	0.99	0.79	1.48	0.85	0.96	0.94	0.92	0.64	0.11	-0.23	-0.34	-0.38
2017	Klein Bonaire	118	11	0.75	-1.09	-0.40	-0.42	-0.42	-0.41	-0.42	-0.42	-0.42	-0.42	-0.43	-0.44	-0.45	-0.48	-0.53
2017	Klein Bonaire	119	11	35.25	4.89	2.74	0.63	0.48	1.03	0.52	0.61	0.59	0.57	0.35	-0.07	-0.36	-0.45	-0.49
2017	Klein Bonaire	120	11	42.81	6.38	3.54	0.87	0.67	1.37	0.73	0.84	0.82	0.80	0.52	-0.02	-0.37	-0.48	-0.51
2017	Klein Bonaire	121	11	25.18	2.46	1.47	0.16	0.07	0.41	0.09	0.15	0.14	0.13	-0.01	-0.27	-0.44	-0.50	-0.52
2017	Klein Bonaire	122	11	29.75	2.40	1.45	0.16	0.06	0.40	0.09	0.14	0.13	0.12	-0.02	-0.28	-0.45	-0.52	-0.56
2017	Klein Bonaire	123	11	13.19	0.01	0.24	-0.39	-0.44	-0.27	-0.43	-0.40	-0.41	-0.41	-0.48	-0.60	-0.69	-0.73	-0.75
2017	Klein Bonaire	124	11	40.12	3.20	1.97	0.14	0.00	0.48	0.04	0.12	0.10	0.09	-0.11	-0.48	-0.72	-0.80	-0.83
2017	Klein Bonaire	125	11	24.95	1.30	1.03	-0.29	-0.39	-0.04	-0.36	-0.31	-0.32	-0.33	-0.47	-0.74	-0.92	-0.98	-1.01
2017	Klein Bonaire	126	11	35.84	1.04	1.25	-0.98	-1.15	-0.56	-1.10	-1.01	-1.03	-1.04	-1.28	-1.73	-2.03	-2.13	-2.17
2017	Klein Bonaire	127	11	22.26	0.81	0.72	-0.35	-0.43	-0.14	-0.40	-0.36	-0.37	-0.38	-0.49	-0.71	-0.85	-0.91	-0.94
2017	Central	24	6	7.83	-0.31	-0.05	-0.29	-0.31	-0.25	-0.31	-0.30	-0.30	-0.30	-0.33	-0.38	-0.41	-0.43	-0.45
2017	Central	25	6	14.82	0.31	0.26	-0.14	-0.16	-0.06	-0.16	-0.14	-0.14	-0.15	-0.19	-0.27	-0.33	-0.36	-0.39

2017	Central	26	6	0.55	-1.50	-0.57	-0.59	-0.59	-0.58	-0.59	-0.59	-0.59	-0.59	-0.60	-0.60	-0.61	-0.63	-0.66
2017	Central	27	6	7.94	-1.49	-0.42	-0.74	-0.76	-0.68	-0.75	-0.74	-0.74	-0.75	-0.78	-0.85	-0.89	-0.91	-0.93
2017	Central	28	6	18.34	1.87	1.11	0.11	0.04	0.30	0.06	0.10	0.09	0.08	-0.02	-0.23	-0.36	-0.42	-0.45
2017	Central	29	6	9.09	0.01	0.21	-0.33	-0.37	-0.23	-0.36	-0.34	-0.34	-0.34	-0.40	-0.51	-0.59	-0.62	-0.65
2017	Central	30	6	0	-0.15	-0.05	-0.05	-0.05	-0.05	-0.05	-0.05	-0.05	-0.05	-0.05	-0.05	-0.05	-0.06	-0.06
2017	Central	31	6	0	-0.02	-0.01	-0.01	-0.01	-0.01	-0.01	-0.01	-0.01	-0.01	-0.01	-0.01	-0.01	-0.01	-0.01
2017	Central	32	6	0.5	-0.07	-0.03	-0.04	-0.04	-0.04	-0.04	-0.04	-0.04	-0.04	-0.04	-0.04	-0.05	-0.05	-0.06
2017	Central	33	6	1.31	-0.58	-0.21	-0.24	-0.24	-0.23	-0.24	-0.24	-0.24	-0.24	-0.25	-0.25	-0.26	-0.27	-0.29
2017	Central	34	6	1.94	-1.33	-0.54	-0.57	-0.58	-0.57	-0.58	-0.58	-0.58	-0.57	-0.58	-0.59	-0.60	-0.62	-0.65
2017	Central	35	6	10.25	-0.17	-0.01	-0.21	-0.23	-0.17	-0.23	-0.22	-0.22	-0.22	-0.24	-0.29	-0.32	-0.34	-0.37
2017	Central	36	6	14.25	-0.85	-0.17	-0.58	-0.61	-0.50	-0.60	-0.59	-0.59	-0.59	-0.64	-0.72	-0.78	-0.81	-0.83
2017	Central	37	6	5.57	-0.02	0.03	-0.11	-0.12	-0.08	-0.11	-0.11	-0.11	-0.11	-0.13	-0.15	-0.18	-0.19	-0.20
2017	Central	38	6	6.54	-1.52	-0.47	-0.67	-0.69	-0.63	-0.68	-0.67	-0.67	-0.68	-0.70	-0.74	-0.77	-0.78	-0.79
2017	Central	39	6	15.17	-0.34	0.10	-0.61	-0.66	-0.47	-0.64	-0.61	-0.62	-0.62	-0.70	-0.84	-0.94	-0.98	-1.00
2017	Central	40	6	12.35	5.59	3.25	0.85	0.67	1.30	0.72	0.82	0.80	0.78	0.53	0.05	-0.26	-0.35	-0.37
2017	Central	41	6	15.48	-0.66	-0.07	-0.63	-0.67	-0.52	-0.66	-0.64	-0.64	-0.64	-0.70	-0.82	-0.90	-0.94	-0.97
2017	Central	42	6	14.86	-0.12	0.17	-0.44	-0.48	-0.32	-0.47	-0.45	-0.45	-0.45	-0.52	-0.64	-0.73	-0.77	-0.80
2017	Central	43	6	5.89	-1.54	-0.49	-0.63	-0.64	-0.60	-0.64	-0.63	-0.63	-0.63	-0.65	-0.68	-0.70	-0.73	-0.76
2017	Central	44	6	2.31	-1.63	-0.57	-0.63	-0.64	-0.62	-0.64	-0.64	-0.64	-0.64	-0.65	-0.66	-0.67	-0.69	-0.71
2017	Central	45	6	5.33	0.55	0.27	0.06	0.04	0.10	0.05	0.06	0.06	0.05	0.03	-0.01	-0.04	-0.05	-0.05
2017	Central	46	6	4.91	-0.42	-0.10	-0.32	-0.34	-0.28	-0.33	-0.32	-0.33	-0.33	-0.35	-0.40	-0.43	-0.45	-0.46
2017	Central	47	6	12.41	0.98	0.56	-0.03	-0.07	0.08	-0.06	-0.04	-0.04	-0.05	-0.11	-0.23	-0.31	-0.34	-0.35
2017	Central	48	6	30.86	8.82	5.17	1.27	0.98	2.01	1.07	1.23	1.20	1.17	0.76	-0.02	-0.53	-0.68	-0.71
2017	Central	49	6	2.42	-1.30	-0.41	-0.49	-0.50	-0.47	-0.50	-0.50	-0.50	-0.50	-0.51	-0.53	-0.55	-0.58	-0.62
2017	Central	50	6	37.34	5.88	3.36	0.66	0.45	1.16	0.51	0.63	0.60	0.58	0.30	-0.24	-0.60	-0.71	-0.74
2017	Central	51	6	5.57	-0.14	0.08	-0.29	-0.31	-0.22	-0.31	-0.29	-0.29	-0.30	-0.34	-0.41	-0.46	-0.48	-0.49
2017	Central	52	6	15.17	0.66	0.45	-0.12	-0.16	-0.01	-0.15	-0.13	-0.13	-0.13	-0.20	-0.31	-0.39	-0.43	-0.45
2017	Central	53	6	23.05	1.67	1.07	0.01	-0.07	0.21	-0.05	-0.01	-0.01	-0.02	-0.14	-0.35	-0.50	-0.56	-0.60
2017	Central	128	6	3.91	-0.37	-0.14	-0.20	-0.20	-0.18	-0.20	-0.20	-0.20	-0.20	-0.20	-0.22	-0.23	-0.24	-0.25
2017	Central	129	6	0.72	-1.05	-0.46	-0.48	-0.48	-0.47	-0.48	-0.48	-0.48	-0.48	-0.48	-0.49	-0.49	-0.51	-0.53
2017	Central	130	6	4.68	-0.82	-0.32	-0.42	-0.42	-0.40	-0.42	-0.42	-0.42	-0.42	-0.43	-0.45	-0.47	-0.49	-0.51
2017	Central	24	11	32.56	2.95	1.74	0.23	0.12	0.52	0.16	0.22	0.21	0.19	0.04	-0.27	-0.46	-0.53	-0.55
2017	Central	25	11	52.33	7.78	4.41	1.00	0.74	1.64	0.82	0.96	0.93	0.91	0.55	-0.14	-0.58	-0.72	-0.75

2017	Central	26	11	29.14	2.82	1.57	-0.01	-0.13	0.29	-0.10	-0.03	-0.04	-0.05	-0.22	-0.54	-0.75	-0.82	-0.85
2017	Central	27	11	16.63	-0.33	0.10	-0.44	-0.48	-0.34	-0.47	-0.45	-0.46	-0.46	-0.52	-0.63	-0.71	-0.76	-0.81
2017	Central	28	11	23.53	1.72	1.13	0.03	-0.05	0.24	-0.03	0.01	0.00	0.00	-0.12	-0.35	-0.50	-0.58	-0.64
2017	Central	29	11	11.99	0.45	0.36	-0.11	-0.15	-0.02	-0.14	-0.12	-0.13	-0.13	-0.18	-0.28	-0.35	-0.39	-0.43
2017	Central	30	11	2.09	-1.66	-0.57	-0.62	-0.63	-0.61	-0.63	-0.63	-0.63	-0.62	-0.63	-0.65	-0.66	-0.68	-0.71
2017	Central	31	11	12.17	0.07	0.12	-0.19	-0.21	-0.13	-0.21	-0.20	-0.20	-0.20	-0.24	-0.30	-0.34	-0.37	-0.40
2017	Central	32	11	14.98	-0.13	0.09	-0.30	-0.33	-0.22	-0.32	-0.31	-0.31	-0.31	-0.35	-0.43	-0.49	-0.53	-0.56
2017	Central	33	11	25.84	1.36	0.96	-0.13	-0.21	0.08	-0.18	-0.14	-0.15	-0.16	-0.27	-0.49	-0.64	-0.71	-0.76
2017	Central	34	11	0.5	-0.65	-0.25	-0.25	-0.25	-0.25	-0.25	-0.26	-0.26	-0.25	-0.26	-0.26	-0.27	-0.28	-0.31
2017	Central	35	11	30.25	1.92	1.28	-0.11	-0.21	0.15	-0.18	-0.12	-0.13	-0.14	-0.29	-0.57	-0.76	-0.82	-0.85
2017	Central	36	11	25.91	2.38	1.25	0.11	0.03	0.33	0.05	0.10	0.09	0.08	-0.04	-0.27	-0.42	-0.48	-0.52
2017	Central	37	11	39.77	5.26	2.65	0.47	0.31	0.89	0.36	0.45	0.43	0.42	0.19	-0.25	-0.54	-0.63	-0.67
2017	Central	38	11	33.18	0.62	0.72	-0.54	-0.64	-0.30	-0.61	-0.56	-0.57	-0.58	-0.71	-0.97	-1.14	-1.20	-1.24
2017	Central	39	11	20.85	0.73	0.65	-0.42	-0.50	-0.22	-0.48	-0.43	-0.44	-0.45	-0.56	-0.78	-0.92	-0.98	-1.02
2017	Central	40	11	34.45	2.00	1.47	-0.34	-0.47	0.00	-0.43	-0.36	-0.37	-0.39	-0.58	-0.94	-1.18	-1.26	-1.29
2017	Central	41	11	14.56	-0.83	-0.11	-0.72	-0.77	-0.61	-0.75	-0.73	-0.74	-0.74	-0.81	-0.93	-1.02	-1.06	-1.10
2017	Central	42	11	19.22	1.47	0.99	-0.16	-0.24	0.06	-0.22	-0.17	-0.18	-0.19	-0.31	-0.55	-0.70	-0.76	-0.80
2017	Central	43	11	25.34	1.98	1.14	-0.10	-0.19	0.14	-0.16	-0.11	-0.12	-0.13	-0.26	-0.51	-0.68	-0.74	-0.78
2017	Central	44	11	23.59	1.26	0.95	-0.43	-0.53	-0.16	-0.50	-0.44	-0.45	-0.46	-0.61	-0.89	-1.07	-1.14	-1.17
2017	Central	45	11	8.52	-0.19	0.04	-0.27	-0.30	-0.21	-0.29	-0.28	-0.28	-0.28	-0.32	-0.39	-0.43	-0.47	-0.50
2017	Central	46	11	17.65	0.34	0.47	-0.49	-0.56	-0.31	-0.54	-0.51	-0.51	-0.52	-0.62	-0.82	-0.95	-1.01	-1.04
2017	Central	47	11	13.22	0.00	0.20	-0.38	-0.43	-0.27	-0.42	-0.39	-0.40	-0.40	-0.46	-0.58	-0.66	-0.70	-0.72
2017	Central	48	11	32.62	2.03	1.36	-0.15	-0.26	0.14	-0.23	-0.17	-0.18	-0.19	-0.35	-0.65	-0.86	-0.93	-0.97
2017	Central	49	11	20.76	2.11	1.42	-0.15	-0.26	0.15	-0.23	-0.17	-0.18	-0.19	-0.36	-0.67	-0.89	-0.97	-1.01
2017	Central	50	11	23.14	1.02	0.89	-0.45	-0.55	-0.20	-0.52	-0.47	-0.48	-0.49	-0.63	-0.90	-1.08	-1.14	-1.17
2017	Central	51	11	30.5	4.43	2.52	0.45	0.30	0.84	0.34	0.43	0.41	0.39	0.18	-0.24	-0.52	-0.61	-0.64
2017	Central	52	11	36.15	5.68	3.41	0.59	0.38	1.12	0.44	0.56	0.54	0.51	0.22	-0.35	-0.72	-0.83	-0.86
2017	Central	53	11	39.96	5.71	3.30	0.53	0.32	1.05	0.38	0.50	0.47	0.45	0.16	-0.40	-0.76	-0.87	-0.91
2017	Central	128	11	21.07	1.43	0.92	-0.03	-0.10	0.15	-0.08	-0.04	-0.05	-0.05	-0.15	-0.35	-0.48	-0.53	-0.56
2017	Central	129	11	16.17	1.33	0.93	-0.16	-0.23	0.05	-0.21	-0.17	-0.18	-0.18	-0.30	-0.52	-0.67	-0.72	-0.76
2017	Central	130	11	13.81	0.69	0.48	-0.09	-0.13	0.02	-0.12	-0.10	-0.10	-0.10	-0.16	-0.28	-0.36	-0.40	-0.43
2017	South	3	6	4.67	-0.91	-0.34	-0.49	-0.50	-0.46	-0.49	-0.49	-0.49	-0.49	-0.51	-0.54	-0.56	-0.58	-0.61
2017	South	4	6	11.08	0.20	0.15	-0.11	-0.13	-0.06	-0.12	-0.11	-0.11	-0.11	-0.14	-0.20	-0.24	-0.26	-0.27

2017	South	5	6	16	-0.35	-0.04	-0.39	-0.41	-0.32	-0.41	-0.39	-0.39	-0.40	-0.44	-0.51	-0.56	-0.59	-0.61
2017	South	6	6	1.49	-0.12	-0.06	-0.07	-0.07	-0.06	-0.07	-0.07	-0.07	-0.07	-0.07	-0.07	-0.08	-0.08	-0.09
2017	South	7	6	2.48	-0.04	-0.02	-0.04	-0.04	-0.04	-0.04	-0.04	-0.04	-0.04	-0.05	-0.05	-0.06	-0.06	-0.07
2017	South	8	6	16.55	0.10	0.18	-0.21	-0.24	-0.13	-0.23	-0.21	-0.22	-0.22	-0.26	-0.34	-0.40	-0.43	-0.45
2017	South	9	6	15.62	3.05	1.65	0.49	0.40	0.71	0.42	0.47	0.46	0.46	0.33	0.10	-0.06	-0.11	-0.12
2017	South	10	6	6.38	2.17	1.25	0.31	0.24	0.49	0.26	0.30	0.29	0.28	0.18	-0.01	-0.14	-0.18	-0.19
2017	South	11	6	0	-0.04	-0.01	-0.01	-0.01	-0.01	-0.01	-0.01	-0.01	-0.01	-0.01	-0.01	-0.01	-0.02	-0.02
2017	South	12	6	7.61	0.10	0.08	-0.05	-0.06	-0.03	-0.06	-0.05	-0.05	-0.05	-0.07	-0.09	-0.11	-0.12	-0.13
2017	South	13	6	6.13	-0.01	0.00	-0.06	-0.06	-0.04	-0.06	-0.06	-0.06	-0.06	-0.07	-0.08	-0.09	-0.10	-0.12
2017	South	15	6	2.8	-2.21	-0.77	-0.83	-0.84	-0.82	-0.84	-0.84	-0.84	-0.83	-0.84	-0.86	-0.87	-0.89	-0.91
2017	South	16	6	4.94	0.16	0.10	0.00	0.00	0.02	0.00	0.00	0.00	0.00	-0.01	-0.03	-0.05	-0.06	-0.07
2017	South	17	6	4.63	-0.02	0.00	-0.07	-0.08	-0.06	-0.08	-0.08	-0.08	-0.07	-0.09	-0.10	-0.12	-0.13	-0.15
2017	South	18	6	2.44	-1.33	-0.55	-0.57	-0.57	-0.56	-0.57	-0.57	-0.57	-0.57	-0.57	-0.57	-0.58	-0.60	-0.62
2017	South	19	6	4.47	0.75	0.55	-0.13	-0.18	0.00	-0.16	-0.14	-0.14	-0.15	-0.22	-0.36	-0.46	-0.50	-0.54
2017	South	20	6	1.65	-0.27	-0.12	-0.14	-0.14	-0.13	-0.14	-0.14	-0.14	-0.14	-0.15	-0.15	-0.16	-0.18	-0.20
2017	South	21	6	20.74	0.99	0.73	-0.19	-0.26	-0.02	-0.24	-0.20	-0.21	-0.22	-0.32	-0.50	-0.63	-0.68	-0.70
2017	South	22	6	1.21	0.09	0.06	0.00	-0.01	0.01	-0.01	-0.01	-0.01	-0.01	-0.01	-0.03	-0.04	-0.04	-0.05
2017	South	23	6	20.82	-0.35	-0.11	-0.21	-0.21	-0.19	-0.21	-0.21	-0.21	-0.21	-0.22	-0.24	-0.26	-0.27	-0.28
2017	South	131	6	18.02	0.63	0.43	-0.12	-0.16	-0.02	-0.15	-0.13	-0.13	-0.14	-0.19	-0.30	-0.38	-0.40	-0.42
2017	South	132	6	21.48	1.34	0.83	0.04	-0.02	0.19	-0.01	0.03	0.02	0.01	-0.07	-0.23	-0.34	-0.38	-0.40
2017	South	133	6	11.01	0.52	0.34	-0.01	-0.04	0.05	-0.03	-0.02	-0.02	-0.02	-0.06	-0.13	-0.18	-0.21	-0.24
2017	South	134	6	11.18	-0.42	-0.07	-0.37	-0.39	-0.31	-0.38	-0.37	-0.37	-0.38	-0.41	-0.47	-0.51	-0.53	-0.55
2017	South	2	11	0.05	-0.10	-0.04	-0.04	-0.04	-0.04	-0.04	-0.04	-0.04	-0.04	-0.04	-0.04	-0.05	-0.05	-0.05
2017	South	3	11	25.66	3.05	1.72	0.36	0.26	0.62	0.29	0.35	0.33	0.32	0.18	-0.09	-0.27	-0.33	-0.35
2017	South	4	11	20.03	1.06	0.70	-0.07	-0.13	0.07	-0.11	-0.08	-0.09	-0.09	-0.17	-0.33	-0.43	-0.46	-0.48
2017	South	5	11	24.47	1.04	0.73	-0.13	-0.20	0.03	-0.18	-0.14	-0.15	-0.16	-0.25	-0.42	-0.53	-0.57	-0.59
2017	South	6	11	25.79	0.70	0.55	-0.23	-0.29	-0.08	-0.27	-0.24	-0.25	-0.25	-0.34	-0.50	-0.60	-0.64	-0.66
2017	South	7	11	30.4	2.43	1.44	0.18	0.08	0.42	0.11	0.16	0.15	0.14	0.01	-0.24	-0.41	-0.47	-0.49
2017	South	8	11	26.1	1.28	0.83	-0.04	-0.10	0.13	-0.08	-0.05	-0.05	-0.06	-0.15	-0.33	-0.44	-0.49	-0.52
2017	South	9	11	28.31	4.76	2.69	0.62	0.47	1.01	0.51	0.60	0.58	0.57	0.35	-0.06	-0.33	-0.42	-0.44
2017	South	10	11	6.79	-0.66	-0.16	-0.39	-0.41	-0.35	-0.40	-0.39	-0.39	-0.39	-0.42	-0.46	-0.49	-0.50	-0.51
2017	South	11	11	9.12	0.50	0.29	0.03	0.01	0.08	0.01	0.03	0.02	0.02	-0.01	-0.06	-0.10	-0.11	-0.12
2017	South	12	11	21.45	1.12	0.80	-0.15	-0.22	0.03	-0.20	-0.16	-0.17	-0.17	-0.27	-0.46	-0.59	-0.63	-0.64

2017	South	13	11	23.5	1.44	0.93	-0.05	-0.12	0.13	-0.10	-0.06	-0.07	-0.08	-0.18	-0.38	-0.51	-0.56	-0.58
2017	South	14	11	10.42	-0.93	-0.26	-0.57	-0.59	-0.51	-0.58	-0.57	-0.57	-0.58	-0.61	-0.67	-0.71	-0.73	-0.74
2017	South	15	11	29.33	2.19	1.34	0.13	0.04	0.36	0.06	0.11	0.10	0.09	-0.04	-0.28	-0.45	-0.51	-0.54
2017	South	16	11	30.56	1.14	0.94	-0.30	-0.39	-0.06	-0.36	-0.32	-0.32	-0.33	-0.47	-0.72	-0.89	-0.96	-1.00
2017	South	17	11	17.13	1.23	0.68	-0.02	-0.07	0.11	-0.05	-0.03	-0.03	-0.04	-0.11	-0.25	-0.35	-0.39	-0.41
2017	South	18	11	12.19	-1.06	-0.22	-0.79	-0.83	-0.68	-0.82	-0.80	-0.80	-0.80	-0.87	-0.98	-1.07	-1.11	-1.15
2017	South	19	11	26.9	1.68	1.17	-0.12	-0.21	0.13	-0.19	-0.14	-0.15	-0.15	-0.29	-0.56	-0.74	-0.82	-0.87
2017	South	20	11	24.08	2.18	1.45	-0.06	-0.17	0.23	-0.14	-0.08	-0.09	-0.10	-0.26	-0.56	-0.77	-0.85	-0.90
2017	South	21	11	18.97	0.70	0.62	-0.27	-0.33	-0.10	-0.32	-0.28	-0.29	-0.29	-0.39	-0.57	-0.70	-0.76	-0.80
2017	South	22	11	28.05	2.90	1.74	0.20	0.09	0.50	0.12	0.19	0.17	0.16	0.00	-0.31	-0.52	-0.59	-0.63
2017	South	23	11	38.84	3.43	2.18	0.07	-0.09	0.47	-0.04	0.04	0.02	0.01	-0.21	-0.64	-0.92	-1.02	-1.06
2017	South	131	11	52.88	2.24	1.41	0.02	-0.09	0.28	-0.06	0.00	-0.01	-0.02	-0.17	-0.45	-0.63	-0.69	-0.70
2017	South	132	11	29.89	2.30	1.29	0.27	0.20	0.47	0.22	0.26	0.25	0.25	0.14	-0.06	-0.20	-0.24	-0.25
2017	South	133	11	49.61	5.56	2.98	0.95	0.80	1.33	0.84	0.93	0.91	0.89	0.68	0.28	0.01	-0.07	-0.08
2017	South	134	11	9.71	-0.38	-0.05	-0.40	-0.42	-0.33	-0.42	-0.40	-0.41	-0.41	-0.45	-0.52	-0.57	-0.59	-0.61

Any use of trade, firm, or product names is for descriptive purposes only and does not imply endorsement by the U.S. Government.
