# Supplementary material for: Reduced Atlantic reef growth past 2 °C warming amplifies sea-level impacts
Source: Nature. 2025 Sep 17;646(8085):619–26. doi: 10.1038/s41586-025-09439-4 (PMC12527930; doi:10.1038/s41586-025-09439-4)
Supplement: Supplementary file 2 — Reporting Summary [file 41586_2025_9439_MOESM2_ESM.pdf]

nature portfolio

Corresponding author(s): C T Perry
Last updated by author(s): Jul 11, 2025

Reporting Summary

Nature Portfolio wishes to improve the reproducibility of the work that we publish. This form provides structure for consistency and transparency in reporting. For further information on Nature Portfolio policies, see our Editorial Policies and the Editorial Policy Checklist.

Statistics

For all statistical analyses, confirm that the following items are present in the figure legend, table legend, main text, or Methods section.

n/a	Confirmed	
☐	☒	The exact sample size (*n*) for each experimental group/condition, given as a discrete number and unit of measurement
☐	☒	A statement on whether measurements were taken from distinct samples or whether the same sample was measured repeatedly
☐	☒	The statistical test(s) used AND whether they are one- or two-sided<br>*Only common tests should be described solely by name; describe more complex techniques in the Methods section.*
☐	☒	A description of all covariates tested
☐	☒	A description of any assumptions or corrections, such as tests of normality and adjustment for multiple comparisons
☐	☒	A full description of the statistical parameters including central tendency (e.g. means) or other basic estimates (e.g. regression coefficient) AND variation (e.g. standard deviation) or associated estimates of uncertainty (e.g. confidence intervals)
☐	☒	For null hypothesis testing, the test statistic (e.g. *F*, *t*, *r*) with confidence intervals, effect sizes, degrees of freedom and *P* value noted<br>*Give P values as exact values whenever suitable.*
☐	☒	For Bayesian analysis, information on the choice of priors and Markov chain Monte Carlo settings
☐	☒	For hierarchical and complex designs, identification of the appropriate level for tests and full reporting of outcomes
☐	☒	Estimates of effect sizes (e.g. Cohen's *d*, Pearson's *r*), indicating how they were calculated

Our web collection on statistics for biologists contains articles on many of the points above.

Software and code

Policy information about availability of computer code

Data collection	Fossil imagery data used to assess reef framework stacking porosity values was compiled from existing collections from across the authorship team. Carbonate budget data reported here were collected using the established ReefBudget protocol (www.exeter.ac.uk/research/projects/geography/reefbudget/) by regional members of the authorship team between 2016 and 2023. Data for comparative analysis to assess the impact of using the new reef stacking porosity values on reef accretion rates was undertaken against data used in a 2018 Nature paper (doi.org/10.1038/s41586-018-0194-z).
Data analysis	Fossil reef imagery data were analysed by importing imagery first into Adobe Illustrator (version 2024) to delineate framework components, then into Adobe Photoshop (version 2024) for conversion to B&W images, and then into ImageJ (Dec 2023 version) to determine the % of framework to non-framework components using thresholding analysis. Analysis of contemporary reef carbonate budget data and of estimated reef accretion potential rates, both today and under future SST and OA effects, was undertaken using the following: R (V4.0.4), CDO (1.9.10), Image J (Dec 2023 version), Microsoft Excel (2024), and GraphPad Prism 10. Computer codes used to produce the projections of coral cover change and SST and OA impacts on coral and coralline calcification, and on substrate bioerosion rates are freely available at https://github.com/ComeauS/Perry_et_al_Caribbean/tree/main. The following packages were used in R: Tidyverse #Data processing and organisation (v2.0.0); Future #For Parallel processing (v1.33.1); Tidync #For reading netcdf files (v0.3.0); Data.table #Data processing and organisation (v1.16.4); Arrow #Write compressed output files (v9.0.0.20); sf #spatial analysis (v1.0-15); Plyr (v1.8.9). Projected rates of reef accretion were compared to projected rates of sea-level rise using projections based on the findings of Chapter 9 of the Working Group 1 contribution to the IPCC Sixth Assessment Report, the Framework for Assessment of Changes To Sea-level (FACTS) and accessed via the NASA Sea Level Projection Tool (https://toolkit.climate.gov/tool/ipcc-ar6-sea-level-projection-tool).

For manuscripts utilizing custom algorithms or software that are central to the research but not yet described in published literature, software must be made available to editors and reviewers. We strongly encourage code deposition in a community repository (e.g. GitHub). See the Nature Portfolio guidelines for submitting code & software for further information.

Data

Policy information about availability of data

All manuscripts must include a data availability statement. This statement should provide the following information, where applicable:

- Accession codes, unique identifiers, or web links for publicly available datasets
- A description of any restrictions on data availability
- For clinical datasets or third party data, please ensure that the statement adheres to our policy

Supplementary information and supporting data is provided as a pdf file. Additional site-specific rate data supporting this publication are openly available from the University of Exeter's institutional repository at: https://doi.org/10.24378/exe.5766.

Research involving human participants, their data, or biological material

Policy information about studies with human participants or human data. See also policy information about sex, gender (identity/presentation), and sexual orientation and race, ethnicity and racism.

Reporting on sex and gender	No human participants or their data or biological material were used in this study.
Reporting on race, ethnicity, or other socially relevant groupings	No human participants or their data or biological material were used in this study.
Population characteristics	No human participants or their data or biological material were used in this study.
Recruitment	No human participants or their data or biological material were used in this study.
Ethics oversight	No human participants or their data or biological material were used in this study.

Note that full information on the approval of the study protocol must also be provided in the manuscript.

Field-specific reporting

Please select the one below that is the best fit for your research. If you are not sure, read the appropriate sections before making your selection.

☐ Life sciences ☐ Behavioural & social sciences ☒ Ecological, evolutionary & environmental sciences

For a reference copy of the document with all sections, see nature.com/documents/nr-reporting-summary-flat.pdf

Ecological, evolutionary & environmental sciences study design

All studies must disclose on these points even when the disclosure is negative.

Study description	This study explores future coral reef growth potential under projected rates of sea-level rise. Specifically, it constrains time points and magnitudes of water depth increases above coral reefs under different Shared Socioeconomic Pathway (SSP) emission scenarios through to 2100.
Research sample	Data on rates of reef carbonate production and erosion were used to describe the carbonate budget state of reefs at discrete reef sites located along the Florida Keys, in the Gulf of Mexico and along the Caribbean coast of Mexico, and from around the island of Bonaire. These data were previously collected by members of the authorship team and provide the basis for the assessments of reef accretion potential through to 2100 we conduct here. Underpinning the reef accretion assessments are data on reef framework stacking porosity values calculated from images collected by the authorship team from fossil reef outcrops in the Caribbean.
Sampling strategy	Carbonate budget data used in this study were previously collected by in-country teams from sites spanning a range of water depths (0.5-12 m depth) at predetermined sampling locations in each region of interest. The central aim of this data collection was to capture data from a range of sites and water depths in each location.
Data collection	All fossil reef imagery used in the analysis of reef framework stacking porosity values was collected by members of the authorship team or as described in SI Table 2. Carbonate budget data used in the main analysis reported here were collected using the established ReefBudget protocol (www.exeter.ac.uk/research/projects/geography/reefbudget/) by regional members of the authorship team between 2016 and 2023. Comparative data analysis to assess the impact of using the new reef stacking porosity values was undertaken against data used in a 2018 Nature paper (doi.org/10.1038/s41586-018-0194-z).
Timing and spatial scale	Underpinning ReefBudget data used in this study as baseline start points for our analysis were collected at different time periods in each location as a function of the timing of the specific projects involved in each region: Florida - 2016 and 2018; Mexico - between 2017 and 2022; Bonaire - 2017.

Data exclusions	The only data excluded from our analysis were any data collected from sites >12 m depth as we deemed these to be not relevant to determining shallow water reef accretion behaviour.
Reproducibility	Data collection sites and water depths are listed in SI Table 8 for each country.
Randomization	ReefBudget data were grouped for each country into geographic sub-regions in each country and then additionally sorted by depth category (0-6m and 6-12 m)
Blinding	Not applicable as data analysis was based on in-field surveys along transect lines.

Did the study involve field work? ☒ Yes ☐ No

Field work, collection and transport

Field conditions	As typical with all marine based in-water surveying, in-water conditions were variable but any day to day variations in conditions are considered unlikely to have had a major influence on the metrics collected along the benthic transects.
Location	In-field survey data were collected from individual reef sites/water depths at locations spanning the three main regions of interest in Florida, Mexico and Bonaire. Data from each site in provided in SI Table 8.
Access & import/export	No import/export issues as all data recorded in-field.
Disturbance	Data collection and surveying used temporary placed transect lines, removed after each survey, and were non -invasive.

Reporting for specific materials, systems and methods

We require information from authors about some types of materials, experimental systems and methods used in many studies. Here, indicate whether each material, system or method listed is relevant to your study. If you are not sure if a list item applies to your research, read the appropriate section before selecting a response.

Materials & experimental systems

n/a	Involved in the study
☒	☐ Antibodies
☒	☐ Eukaryotic cell lines
☒	☐ Palaeontology and archaeology
☒	☐ Animals and other organisms
☒	☐ Clinical data
☒	☐ Dual use research of concern
☒	☐ Plants

Methods

n/a	Involved in the study
☒	☐ ChIP-seq
☒	☐ Flow cytometry
☒	☐ MRI-based neuroimaging

Plants

Seed stocks	Not used
Novel plant genotypes	Not used
Authentication	Not used
